# Foliar spray of prohexadione-calcium improves the adaptability of mung bean to saline-alkali stress

**DOI:** 10.3389/fpls.2025.1681992

**Published:** 2025-10-24

**Authors:** Xilong Liang, Yanlei Dong, Qiuyu Jiang, Xue Hou, Yang Song, Fengzhou Zhao, Yu Sun, Haipeng Jiang, Shumei Fang, Qingyan Wang

**Affiliations:** ^1^ College of Agriculture, Heilongjiang Bayi Agricultural University, Daqing, China; ^2^ Heilongjiang Plant Growth Regulator Engineering Technology Research Center, Daqing, China; ^3^ College of Life Science and Biotechnology, Heilongjiang Bayi Agricultural University, Daqing, China

**Keywords:** mung bean, saline-alkali stress, prohexadione-calcium, physiological and molecular mechanisms, transcriptomics

## Abstract

**Introduction:**

Saline-alkali soils are a major constraint to mung bean cultivation and extension, and prohexadione-calcium (Pro-Ca) can enhance plant tolerance to saline-alkali stress.

**Methods:**

In order to explore the regulatory effect and mechanism of Pro-Ca on mung bean growth under saline-alkali stress, the morphology, ultrastructure, physiological indicators, and gene expression were measured in this study.

**Results:**

The results indicate that Pro-Ca can improve the adaptability of mung bean to saline-alkali stress. Specifically, it manifests as increasing dry matter accumulation, protecting the structural integrity and quantity of organelles such as chloroplasts and mitochondria, enhancing photosynthetic capacity, increasing antioxidant enzyme activity and the content of osmoregulatory substances. These changes may be related to the enhanced expression of calcium signal transmission and the synthesis of nitric oxide (NO), polyamines and jasmonic acid in the root system under saline-alkali stress.

**Discussion:**

Our findings partially explain the physiological and molecular mechanisms by which Pro-Ca enhances the tolerance and adaptability of mung bean plants to saline-alkali stress. This may become an effective strategy for the utilization of saline-alkali soil.

## 1 Introduction

Mung bean (*Vigna radiata*) is a globally cultivated crop with high nutritional and medicinal value. It contains 20-25% protein, 55-65% carbohydrates, and 7% dietary fiber, along with essential vitamins, minerals, and bioactive compounds such as polyphenols, polysaccharides, and peptides (Gan et al., 2017; Nawaz et al., 2022; Xu et al., 2021). Extensive research has demonstrated its therapeutic potential, including anti-diabetic, anti-hypertensive, hypolipidemic, anti-cancer, and hepatoprotective effects, as well as immune-modulatory and anti-melanogenic properties (Karami et al., 2025; Ketha and Gudipati, 2018; Yao et al., 2016). Due to its dual benefits as a functional food and a source of medicinal compounds, mung bean products are in high demand in global markets.

As a short-duration crop (75–90 days) with strong nitrogen-fixing capacity, it plays a crucial role in sustainable cropping systems (Favero et al., 2021). Farmers frequently incorporate it in rotation with cereals or as an intercrop with cash crops to improve soil health and enhance overall productivity (Gong et al., 2020, 2024; Kawasaki et al., 2025). Globally, mung bean is cultivated on over 7 million hectares, primarily in Asia, America, Australia and Africa (Dahiya et al., 2015; Nair and Schreinemachers, 2020). However, these regions are suffering from increasingly severe soil salinization due to climate change (Eswar et al., 2021; Hassani et al., 2021). Mung bean was also reported a salt sensitive crop (Ashraf and Rasul, 1988), with actual yields typically only reaching 1/5-1/6 of its theoretical potential (Nair et al., 2019). Soil saline-alkalization has become one of the major limiting factors for mung bean production in the world (HanumanthaRao et al., 2016).

Soil salt-alkalization exerts dual stress effects on plant cells including salt and alkali. Salt stress mainly originates from neutral salts (NaCl and Na_2_SO_4_), where excessive Na^+^ influx directly disrupts cellular metabolism. Concurrently, decreased extracellular osmotic potential induces cellular dehydration. The alkaline stress component (NaHCO_3_, Na_2_CO_3_) elevates soil pH, destabilizing intracellular pH homeostasis and impairing protein/enzyme functionality (Peng et al., 2025; Ye et al., 2021; Zhang et al., 2017). Studies demonstrate that combined saline-alkali stress causes more severe physiological damage than either stress alone, including chlorophyll fluorescence, antioxidant enzymes, osmolytes (Chen et al., 2017; Jia et al., 2019; Wang et al., 2017), which are crucial for enhancing crop adaptation to saline-alkali stress (Araújo et al., 2014; Rahim et al., 2025). Plants can adapt to saline-alkali stress to some extent through morphological modifications, physiological homeostasis maintenance, and molecular regulation (Fang et al., 2021; Hasegawa et al., 2000). However, most crops possess limited self-adjustment capacity. Therefore, people have attempted to enhance the tolerance through various means, including QTL/genes, transgenics, microbial inoculants, priming agents (Araújo et al., 2014; EI Moukhtari et al., 2024; Nurbekova et al., 2024). Currently, various priming agents, including plant growth regulators, have been applied to enhance plant saline-alkali tolerance owing to their advantages of fast action and high cost effectiveness (Karnwal et al., 2024; Zulfiqar et al., 2022).

Prohexadione-calcium (Pro-Ca, 3,5-dioxo-4-propionylcyclohexanecarboxylic acid calcium) is an exogenous plant growth regulator that inhibits active gibberellin GA1 biosynthesis (Brown et al., 1997; Kamiya et al., 1991). GA1 mainly exists in vegetative organs, consequently, the application of Pro-Ca can reduce vegetative growth (Medjdoub et al., 2004). Research on rice demonstrated that Pro-Ca reduced internode, stem, and panicle length, while increasing stem strength, which is a morphological improvement that enhances stress adaptation (Kim et al., 2007). Feng et al. (2021) and Yu et al. (2025) demonstrated that Pro-Ca application enhanced saline-alkali tolerance and yield in soybeans by improving photosynthesis, antioxidant capacity, and ion homeostasis. Study on grapes indicated that Pro-Ca optimized fruit quality by regulating hormone balance, sugar-acid metabolism, and enzyme activity (Li et al., 2024). Notably, Pro-Ca exhibits excellent environmental safety, rapidly degrading to tricarballylic acid (a natural plant metabolite) in plants and mineralizing to CO_2_ in soil (half-life <7 days) without leaving harmful residues (Evans et al., 1999). Given these benefits, applying Pro-Ca to improve saline-alkali stress tolerance in mung bean represents a viable and eco-friendly strategy for increasing yield. Our previous work has determined the optimal Pro-Ca spray dose for alleviating saline-alkali stress in mung bean seedling (Hou et al., 2022), but further research is needed to understand the physiological and molecular mechanism.In this work, we identified the alleviating effect of Pro-Ca on saline-alkali stress in mung bean seedlings and explored its underlying physiological and molecular mechanisms The findings provide alternative solutions for the agricultural utilization of saline-alkali land and contribute to understanding the regulatory network of calcium signaling, NO signaling, and phytohormone in plant stress response, providing potential targets for molecular breeding of saline-alkali-tolerant mung bean.

## 2 Material and methods

### 2.1 Experimental materials and conditions

Two mung bean cultivars, Lvfeng2 (LF2, saline-alkali tolerant) and Lvfeng5 (LF5, saline-alkali sensitive), were donated by the Qiqihar Branch of Heilongjiang Academy of Agricultural Sciences. The seeds with full grain and uniform shape were selected and sterilized with 75% ethanol, then rinsed with sterile water for 3 times. They were grown in plastic pots (17-cm diameter × 15-cm deep) containing 2: 1 volume mixture of soil and vermiculite. The soil texture is loose, with organic matter of 3.76%, alkaline hydrolyzed nitrogen of 150.28 mg/kg, available phosphorus of 151.11 mg/kg, available potassium of 368.46 mg/kg, and pH of 6.85. Each pot contains 2 L of substrate. Four plants were maintained per pot after thinning. The pots were randomly placed in a greenhouse. Arrange 30 pots per square meter with 1–2 cm distance between each pot, and place a protective row of pots around the entire arrangement. Plants were grown at day/night atmospheric temperature of 23-28°C and 20-25°C, and with a light intensity of 18,000 lux. Pro-Ca (5%) was provided by Sichuan Guoguang Agrochemical Co., Ltd.

### 2.2 Experiment treatment

Seedlings were subjected to saline-alkali stress and Pro-Ca treatment at the first compound leaf stage. The mixed saline-alkali solution simulates the composition and content of typical saline-alkali soils (NaCl: Na_2_SO_4_: Na_2_CO_3_: NaHCO_3_ = 1:9:9:1, pH=8.5 ± 0.1) with 150 mmol/L concentration (Hou et al., 2022). The experiment comprised four treatment groups: (1) Control (CK): irrigated with water and sprayed with distilled water; (2) Pro-Ca: irrigated with water and sprayed with Pro-Ca solution; (3) SA: irrigated with saline-alkali solution and sprayed with distilled water; (4) SA+Pro-Ca: irrigated with saline-alkali solution and sprayed with Pro-Ca solution. The irrigation volume of saline-alkali solution and control water is 300 mL per pot. The 100 mg/L Pro-Ca solution (Hou et al., 2022) was sprayed evenly on the leaves at a rate of 2 mL per pot. A manual sprayer with an ultra-fine atomizing nozzle was used to spray uniform, fine droplets that easily adhered to the leaf surfaces. Each time, 10 mL of solution was loaded into the sprayer and evenly distributed over five pots. Each treatment consisted of sixty replicate pots.

### 2.3 Determination of shoot and root growth

Plants were sampled at 5, 10, 15, 20, and 25d after treatment, and three pots were taken for each group. The aboveground and underground parts were separated. Shoot height was measured by a ruler and stem diameter were determined with a digital caliper. Total root length, volume, surface area, average diameter, and number of tips were analyzed by a root scanner with WinRHIZO analysis software. The plant samples were dried to a constant weight at 80°C after 105°C for 30min. The dry weights were measured with an electronic analytical balance.

### 2.4 Observation of the ultrastructure

After 20d of treatment, fresh leaves at the same node position and root tips of each treatment were sampled. The leaves were cut into rectangles with a length of 3mm and width of 1mm, which were fixed with cold 2.5% glutaraldehyde (pH 7.5), and suctioned to the bottom of the container. After fixation for one week, the samples were rinsed thoroughly phosphate buffer (0.1 M, pH 6.8), fixed with 1% osmium fixing solution for 2.5 hours, and then rinsed thoroughly with 0.1 M phosphate buffer (0.1 M, pH 6.8) to complete the fixation process. The samples were sequentially dehydrated with 50%, 70%, and 90% ethanol, dehydrated twice with 100% ethanol, and then dehydrated with a 1:1 mixture of 100% ethanol and 100% acetone for 10min. The above process was performed in a 4°C refrigerator. Finally, the dehydration process finished after the samples were soaked in pure acetone at room temperature for 5min. The samples were immersed in a mixture of 100% acetone and Epon812 epoxy resin (1:1), polymerized by heating, and double-stained with uranium acetate/lead nitrate after slicing. Transmission electron microscope (TEM) was used to observe the ultrastructure of samples.

### 2.5 Photosynthetic pigment content

The first fully unfolded compound leaf at the top and other leaves above it were collected on days 5, 10, 15, 20, and 25 after treatment. Three leaves pooled into one biological repeat. Plants with diseased or damaged leaves are discarded. The chlorophyll content was determined by ethanol extraction method. Fresh leaves (approximately 0.2g) cut into 2mm pieces were extracted with 95% (v/v) ethanol and kept at dark for 24h until the tissues became completely white. The absorbances at 665, 649, and 470 nm of the supernatants were determined using a spectrophotometer. Chlorophyll a (Ca), chlorophyll b (Cb) and carotenoid (Caro) contents were calculated using the published formula (Lichtenthaler and Wellburn, 1983): Ca=13.95*A_665_-6.88*A_649_; Cb=24.96*A_649_-7.32*A_665_; Caro=(1000*A_470_-2.05*Ca-114.8*Cb)/245.

### 2.6 Photosynthetic parameters

The first fully unfolded compound leaf at the top was selected for measuring photosynthetic parameters using LI-6400 portable photosynthesis instrument (Li-Cor Company, USA), including net photosynthetic rate (Pn), stomatal conductance (Gs), intercellular CO_2_ concentration (Ci) and transpiration rate (Tr). Plants with diseased or damaged leaves were discarded.

### 2.7 Osmolyte contents

The first fully unfolded compound leaf at the top and other leaves above it were collected on days 5, 10, 15, 20, and 25 after treatment. Three leaves pooled into one biological repeat. Plants with diseased or damaged leaves were discarded. Leaves were ground to powder with liquid nitrogen. Soluble sugar, soluble protein and proline were extracted and quantified through the methods of anthrone, Bradford and ninhydrin, respectively. Three biological replicates were sampled and measured, and three plants were collected per replicate.

### 2.8 Antioxidant enzyme activity and membrane lipid peroxidation

The first fully unfolded compound leaf at the top and other leaves above it were collected on days 5, 10, 15, 20, and 25 after treatment. Three leaves pooled into one biological repeat. Plants with diseased or damaged leaves are discarded. Fresh leaves were quickly ground to powder with liquid nitrogen. SOD, POD and CAT activities were determined using the methods of nitroblue tetrazolium (NBT), guaiacol and hydrogen peroxide, respectively. The thiobarbituric acid (TBA) test was used to determine MDA content. Three biological replicates were sampled and measured, and three plants were collected per replicate.

### 2.9 RNA-seq and differentially expression genes analysis

Plant leaves on the 24h of saline-alkali and Pro-Ca treatments were collected, snap-frozen in liquid nitrogen immediately and stored at -80°C until they were shipped in dry ice to IgenecodeBiotech Co., Ltd. (Beijing, China) for transcriptome sequencing. Each treatment includes three biological replicates. High-quality clean reads were generated from filtering raw data (reads with adapters, reads containing unknown bases and nitrogen content higher than 5%, low-quality reads). HISAT (Hierarchical Indexing for Spliced Alignment of Transcripts) software (v2.0.4) (Kim et al., 2015) was used for the alignment of clean reads for each sample with the mung bean reference genome (https://www.ncbi.nlm.nih.gov/genome/?term=Vigna+radiata). The data are presented in **Supplementary Table 1**. Quantitative analysis is conducted using the featureCounts tool in Subread software (Liao et al., 2014). Following the quantitative analysis of each sample, the data are merged to create an expression array for all samples (Garber et al., 2011). Differential expression analysis was completed by DESeq2 (Love et al., 2014). The normalized readcounts are subjected to a negative binomial distribution model to calculate p-values, followed by multiple hypothesis testing correction to generate the FDR (False Discovery Rate; usually Padj), thereby controlling the false positive rate (Young et al., 2010). The filter threshold was |log2FoldChange| ≥ 1 and Padj value < 0.05. Functional enrichment analysis of DEGs was conducted using clusterProfiler soft in GO (Gene Ontology) and KEGG (Kyoto Encyclopedia of Genes and Genomes) databases. The enrichment results are shown in **Supplementary Table 2**. Padj value < 0.05 means significantly enrichment. The raw data has been deposited in NCBI (BioProject accession number PRJNA1337185).

### 2.10 Data analysis

The experimental data were subjected to one-way analysis of variance using SPSS software (version 22.0, SPSS Inc., USA). P value < 0.05 was considered statistically significant. In addition, morphological data were also statistically analyzed using two-way analysis of variance using SPSS software (version 22.0, SPSS Inc., USA).

## 3 Results

### 3.1 Foliar spray of Pro-Ca promoted shoot and root growth under saline-alkali stress

**Figure 1:**
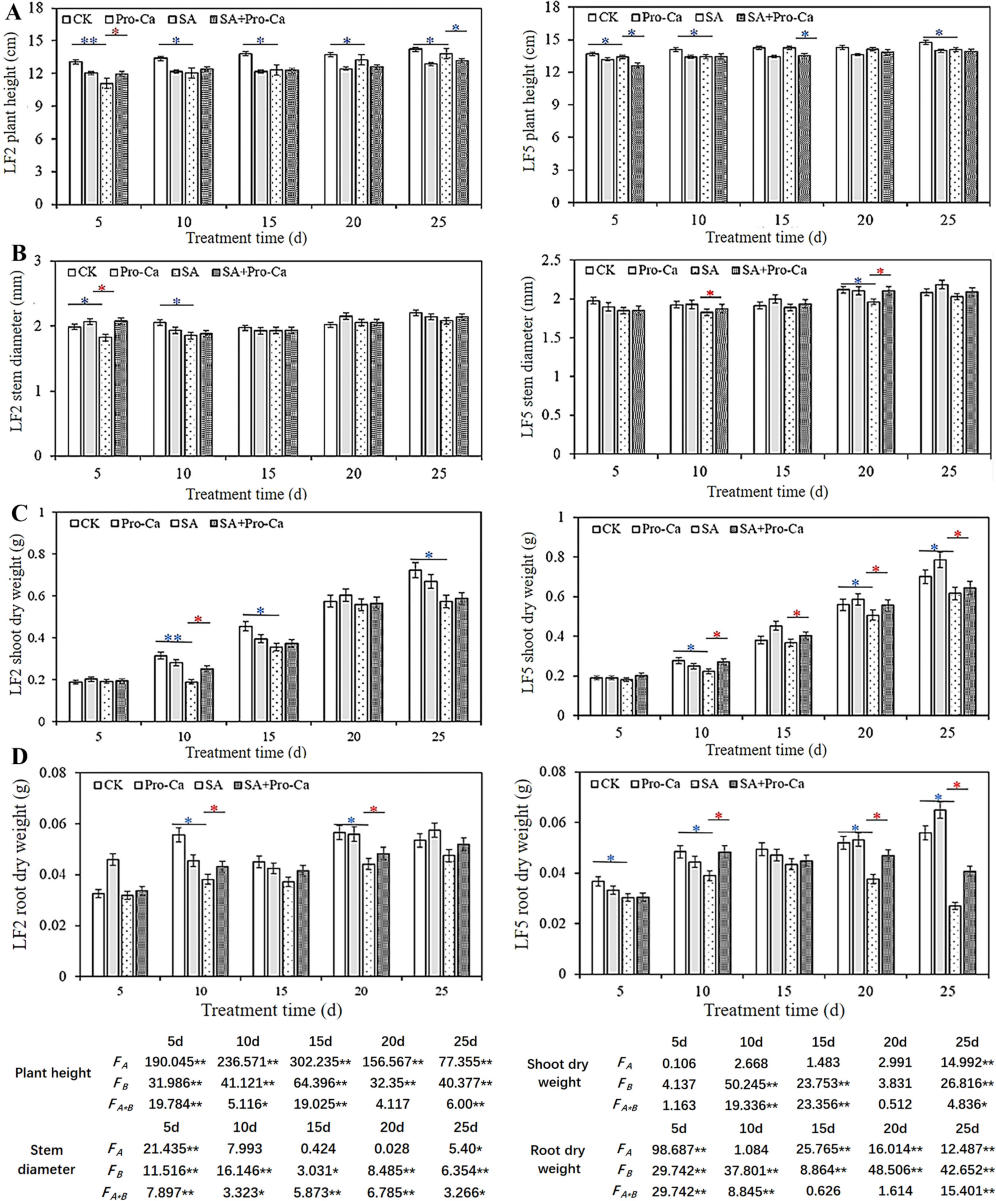
Effects of Pro-Ca on plant growth of mung bean seedlings under saline-alkali stress. CK, Control; Pro-Ca, Pro-Ca spraying; SA, saline-alkali stress; SA+Pro-Ca, Pro-Ca spraying under saline-alkali stress. LF2 means Lvfeng2. LF5 means Lvfeng 5. F_A_ means analysis of variance among varieties, F_B_ means analysis of variance among treatments. * indicates significant difference (P<0.05); ** indicates extremely significant difference (P<0.01). Blue indicates inhibition; Red indicates promotion. The same as below.

As shown in **Figure 1A**, Compared with CK, saline-alkali stress decreased significantly the plant height in both varieties of mung bean by 2.94%-15.11% in LF2, and by 0.14%-4.62% in LF5. After spraying Pro-Ca, the plant height in LF2 increased first and then decreased significantly by 4.70% on the 25th day, while there was similar plant height in LF5 between SA and SA+Pro-Ca group at 25 days. Saline-alkali stress reduced the stem diameter of mung bean plants (**Figure 1B**). After spraying Pro-Ca, the stem diameters of both varieties increased to varying degrees. At 5 days, the stem diameter increased significantly by 12.09% in LF2; At 20 days, the stem diameter increased significantly by 6.77% in LF5, indicating that Pro-Ca can increase stem diameter to some extent under saline-alkali stress.

Saline alkali stress inhibited the accumulation of aboveground biomass in mung bean plants (**Figure 1C**). After spraying Pro-Ca, the shoot dry weight of both varieties of mung beans increased to varying degrees compared with that of the SA group. Among them, the SA+Pro-Ca group in LF2 significantly increased by 25.04% on the 10th day compared to SA; in LF5 variety, SA+Pro-Ca treatment increased significantly the shoot dry weight by 17.77%, 8.63%, 8.78%, and 4.37% on the 10th, 15th, 20th, and 25th days, respectively compared to SA group. This indicates that Pro-Ca spraying can alleviate the inhibition of saline-alkali stress on the accumulation of aboveground matter of the two mung bean varieties, especially for LF5 with a sustained effect. The root growth in both mung bean varieties was inhibited by saline-alkali stress (**Figure 1D**). After spraying with Pro-Ca, the dry weight of the two mung bean varieties increased to varying degrees compared with that of SA. Among them, the SA+Pro-Ca group in LF2 increased significantly by 11.39% and 8.62% respectively on the 10th and 20th days, and in LF5 increased significantly by 19.31%, 19.75% and 33.74% respectively on the 5th, 20th and 25th days, indicating that Pro-Ca can promote the root matter accumulation of mung bean, and has a greater impact on LF5. Root scanning results showed that after spraying Pro-Ca under saline-alkali stress, the root length (8.12%-42.06%), root diameter (3.75%-35.56%), root volume (36.47%-77.32%), root surface area (30.94%-47.20%), and root tip number (14.75%-105.89%) of both varieties of mung beans increased from days 5 to days 25 (**Table 1**), demonstrating that Pro-Ca promoted root development of mung bean plants under saline-alkali stress, thereby contributing to biomass accumulation.

**Table 1:** Effects of Pro-Ca on the root system of mung bean under saline-alkali stress.

Times (d)	Treatments	Root total length(mm)	Root average diam(mm)	Root surface area(cm^2^)	Root volume(cm^3^)	Root tips
5	CK	421.3 ± 59.0ab	0.30 ± 0.01a	37.0± 5.6ab	0.26 ± 0.05ab	1134.5± 77.5ac
	Pro-Ca	519.4 ± 91.0a	0.28 ± 0.01b	46.3± 7.9a	0.33 ± 0.06a	1349.6 ± 264.6ab
	SA	335.6 ± 38.1b	0.28 ± 0.02b	31.7± 7.1b	0.24 ± 0.05b	861.5 ± 128.2bc
	SA+Pro-Ca	476.7 ± 61.1a	0.31 ± 0.01a	46.7± 5.4a	0.36 ± 0.04a	1695.8 ± 357.8a
10	CK	528.4 ± 57.3ab	0.38 ± 0.02a	61.3 ± 4.7ab	0.60 ± 0.05ab	1584.3 ± 323.2a
	Pro-Ca	623.3 ± 62.1a	0.33 ± 0.03ab	64.8± 9.8ab	0.54 ± 0.07b	1569.4 ± 410.1a
	SA	505.8 ± 47.0b	0.35 ± 0.02a	51.7± 9.3ab	0.51 ± 0.09b	1363.3 ± 269.3ab
	SA+Pro-Ca	641.6 ± 72.0a	0.37 ± 0.01a	74.8± 7.3a	0.70 ± 0.06a	1703.4 ± 334.4a
15	CK	613.6 ± 100.9b	0.29 ± 0.01a	54.8± 7.9b	0.39 ± 0.06ab	1302.8 ± 164.3ab
	Pro-Ca	945.7 ± 287.8a	0.29 ± 0.01a	84.7 ± 26.3a	0.61 ± 0.20a	1824.3 ± 611.3a
	SA	438.3± 49.5bc	0.29 ± 0.02a	40.3 ± 8.5bc	0.30 ± 0.08b	1071.5± 85.2bc
	SA+Pro-Ca	564.8± 66.1b	0.30 ± 0.01a	53.5± 6.0b	0.40 ± 0.05ab	1229.5± 75.9b
20	CK	715.7 ± 195.0a	0.41 ± 0.03a	91.2 ± 18.6a	0.94 ± 0.13a	2223.6 ± 212.6a
	Pro-Ca	772.1 ± 164.7a	0.37 ± 0.02a	90.5 ± 15.5a	0.85 ± 0.11ab	2293.0 ± 230.1a
	SA	494.9± 60.2b	0.35 ± 0.06a	57.7 ± 16.7b	0.53 ± 0.27b	1363.8 ± 291.2c
	SA+Pro-Ca	535.1± 40.6b	0.48 ± 0.02a	78.0± 5.7ab	0.95 ± 0.08a	1905.6± 86.6ab
25	CK	855.5 ± 88.2a	0.41 ± 0.02a	97.1 ± 12.6a	0.88 ± 0.13a	3127.0 ± 654.6ab
	Pro-Ca	877.2 ± 109.2a	0.35 ± 0.01b	96.0 ± 11.7a	0.84 ± 0.11a	4886.4 ± 295.7a
	SA	583.7± 80.3b	0.31 ± 0.02c	63.3 ± 11.1b	0.57 ± 0.09b	2249.3 ± 359.5b
	SA+Pro-Ca	769.7 ± 102.3a	0.38 ± 0.01ab	90.6 ± 10.8a	0.85 ± 0.09a	4630.9 ± 930.6a

The two-way analysis of variation (**Figure 1**) showed that there were significant differences (F_A_) in the growth of two mung beans varieties, especially in plant height and root dry weight; Saline-alkali stress and Pro-Ca treatment have significant effects on the growth of mung beans (F_B_), especially on plant height, stem diameter, and root dry weight. The interaction between variety and treatment has the greatest impact on stem thickness (F_A*B_). On the 25th day, the four indicators of plant height, stem diameter, shoot dry weight, and root dry weight showed significant differences among species, treatments, and their interactions, indicating that LF2 and LF5 varieties had significantly different responses to saline-alkali stress and Pro-Ca treatment. Saline-alkali stress and Pro-Ca treatment had significant effects on both varieties, and there was a significant interaction effect between varieties and treatments on mung bean growth.

### 3.2 Pro-Ca protected organelles from damage resulted from saline-alkali stress

**Figure 2:**
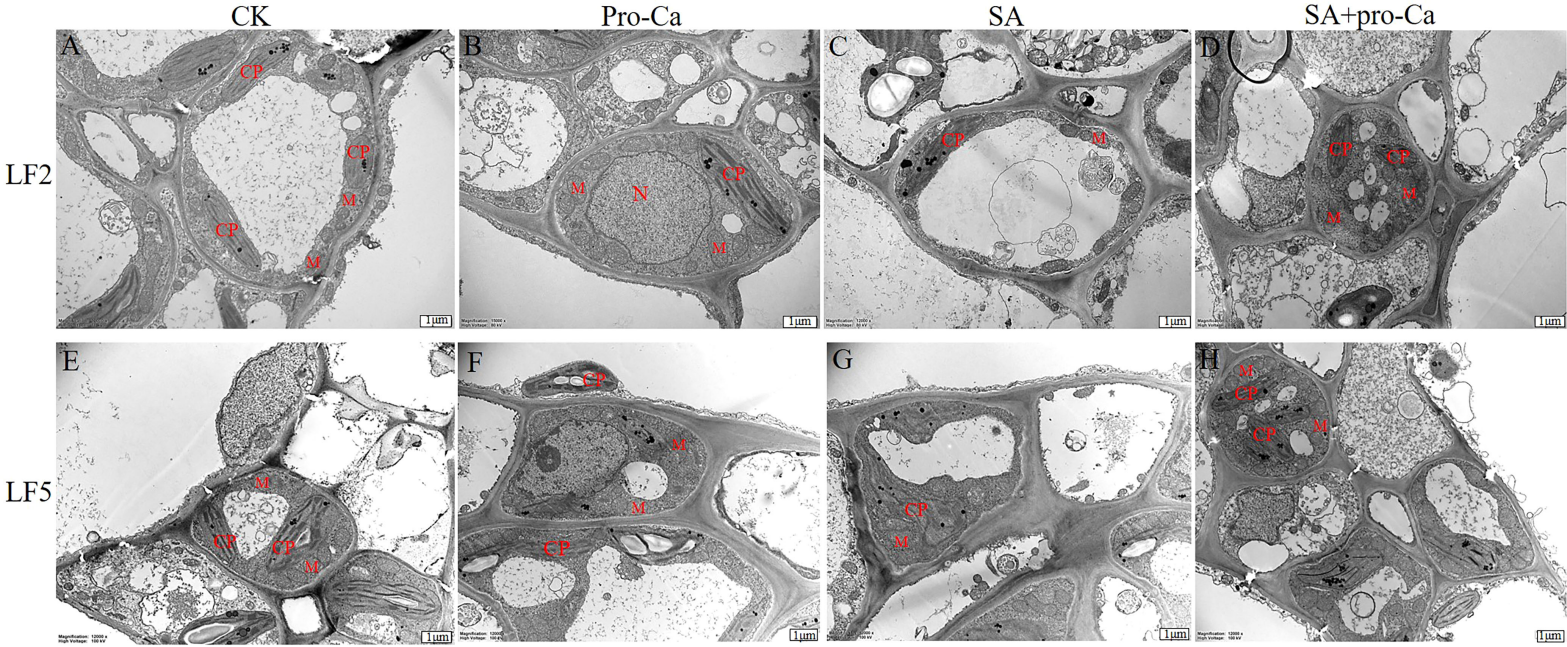
Effects of Pro-Ca on the microstructure of mung bean leaves under saline-alkali stress. CP, Chloroplast; M, Mitochondria.

The ultrastructural changes in the leaf cells are shown in **Figure 2**. There was little difference between the CK and Pro-Ca treatments in LF2 and LF5 (**Figures 2A, B, E, F**). They all contained a number of spindle-shaped chloroplasts close to the cell wall. The double-layer membrane structure of the chloroplast is complete and clear; the grana lamella is smooth and orderly, and is approximately parallel to the long axis of the chloroplast. Mitochondria are elliptically or spherically distributed around the chloroplast, and a small amount of osmophilic granules can be observed.

Under saline-alkali stress, the ultrastructure of leaf cells changed in both LF2 and LF5 (**Figures 2C, D, G, H**). Chloroplasts swelled significantly. Grana lamellae were loose, indistinct, distorted and disordered. The number of mitochondria decreased and the number of osmophilic granules increased. Application of exogenous Pro-Ca protected the chloroplast structural integrity. The number of mitochondria also increased. Grana lamellae arranged more ordered and osmophilic granules were reduced, suggesting that Pro-Ca could protect the ultrastructure of mung bean leaves.

**Figure 3:**
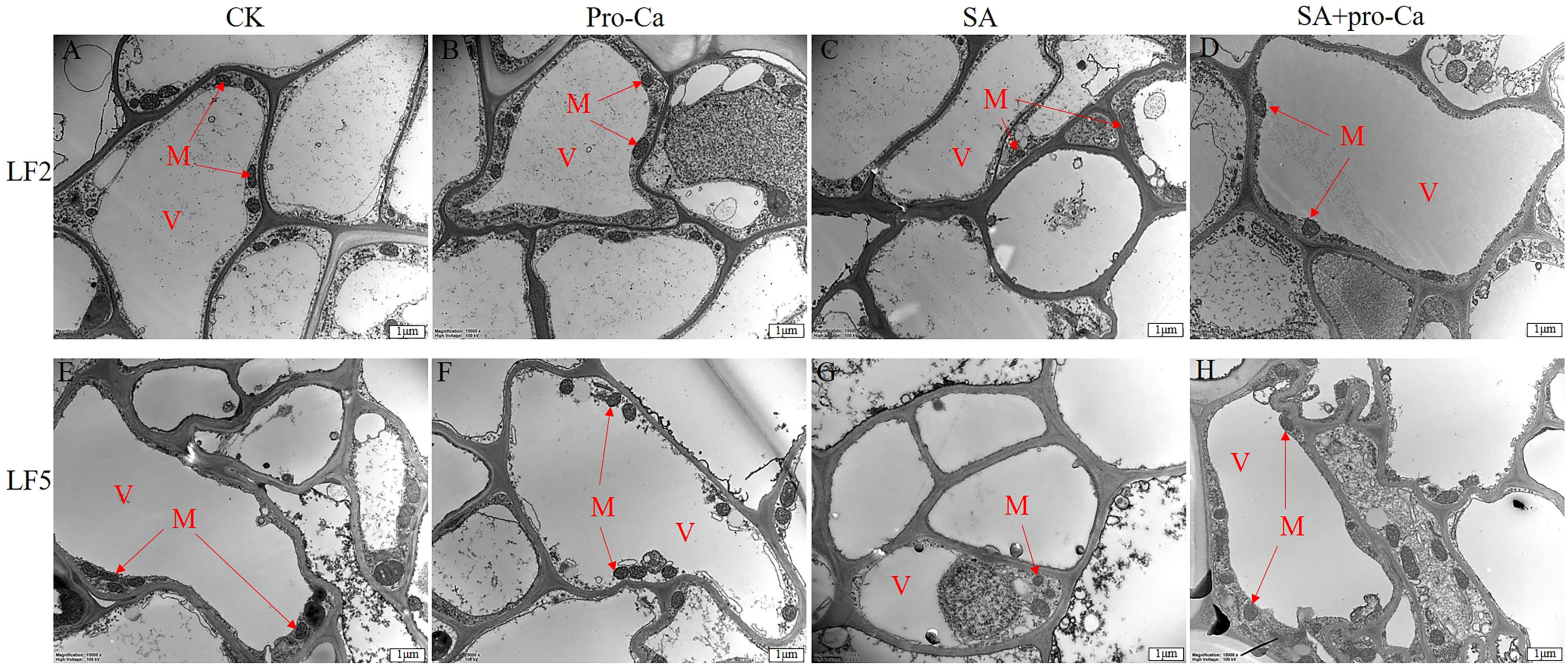
Effects of Pro-Ca on the microstructure of mung bean root apical cells under saline-alkali stress. V, Vacuole; M, Mitochondria.

The subcellular structural changes of root apical cells are mainly reflected in the number of mitochondria. As shown in **Figure 3**, the CK, Pro-Ca and SA+Pro-Ca groups all had a large number of mitochondria, while the SA group had a small number. This suggests that saline-alkali stress can inhibit mitochondrial proliferation, while the spraying of Pro-Ca can alleviate the inhibitory effect.

### 3.3 Pro-Ca enhanced photosynthesis of mung bean leaves under saline-alkali stress

The effects of saline-alkali and Pro-Ca treatments on the chlorophyll content in the two mung bean varieties are shown in **Table 2**. As shown in **Table 2**, the total chlorophyll content of both varieties of mung beans was reduced under saline-alkali stress, whereas it increased after exogenous application of Pro-Ca. At 15, 20, and 25d of SA+Pro-Ca treatment, the total chlorophyll content increased significantly by 139.60%, 68.45%, and 43.66% in LF2 and 47.50%, 82.52%, and 115.53% in LF5 compared to the SA treatment, respectively. This indicates that exogenous application of Pro-Ca can alleviate the reduction in chlorophyll content in mung bean leaves caused by saline-alkali stress.

**Table 2:** Effects of Pro-Ca on the chlorophyll content of LF2 and LF5 under saline-alkali stress.

Days after treatment (d)	Treatments	LF2 (mg/g FW)	LF5 (mg/g FW)
5	CK	1.67 ± 0.12b	1.51 ± 0.08b
	Pro-Ca	1.54 ± 0.31b	1.55 ± 0.44b
	SA	2.72 ± 0.49a	2.13 ± 0.42a
	SA+ Pro-Ca	1.50 ± 0.16b	1.85 ± 0.39ab
10	CK	1.84 ± 0.09a	1.65 ± 0.12a
	Pro-Ca	2.06 ± 0.26a	1.70 ± 0.19a
	SA	1.38 ± 0.27b	1.70 ± 0.15a
	SA+ Pro-Ca	1.66 ± 0.23ab	1.61 ± 0.06a
15	CK	2.02 ± 0.11a	1.72 ± 0.15b
	Pro-Ca	2.23 ± 0.39a	2.07 ± 0.16ab
	SA	1.01 ± 0.17b	1.66 ± 0.07c
	SA+ Pro-Ca	2.42 ± 0.38a	2.45 ± 0.32a
20	CK	1.80 ± 0.15b	2.19 ± 0.46a
	Pro-Ca	2.65 ± 0.26a	2.47 ± 0.25a
	SA	1.68 ± 0.21b	1.43 ± 0.18b
	SA+ Pro-Ca	2.83 ± 0.39a	2.61 ± 0.05a
25	CK	2.70 ± 0.39ab	2.49 ± 0.26a
	Pro-Ca	2.78 ± 0.30a	2.25 ± 0.35a
	SA	1.42 ± 0.28b	1.03 ± 0.11b
	SA+ Pro-Ca	2.04 ± 0.13a	2.22 ± 0.31a

**Figure 4:**
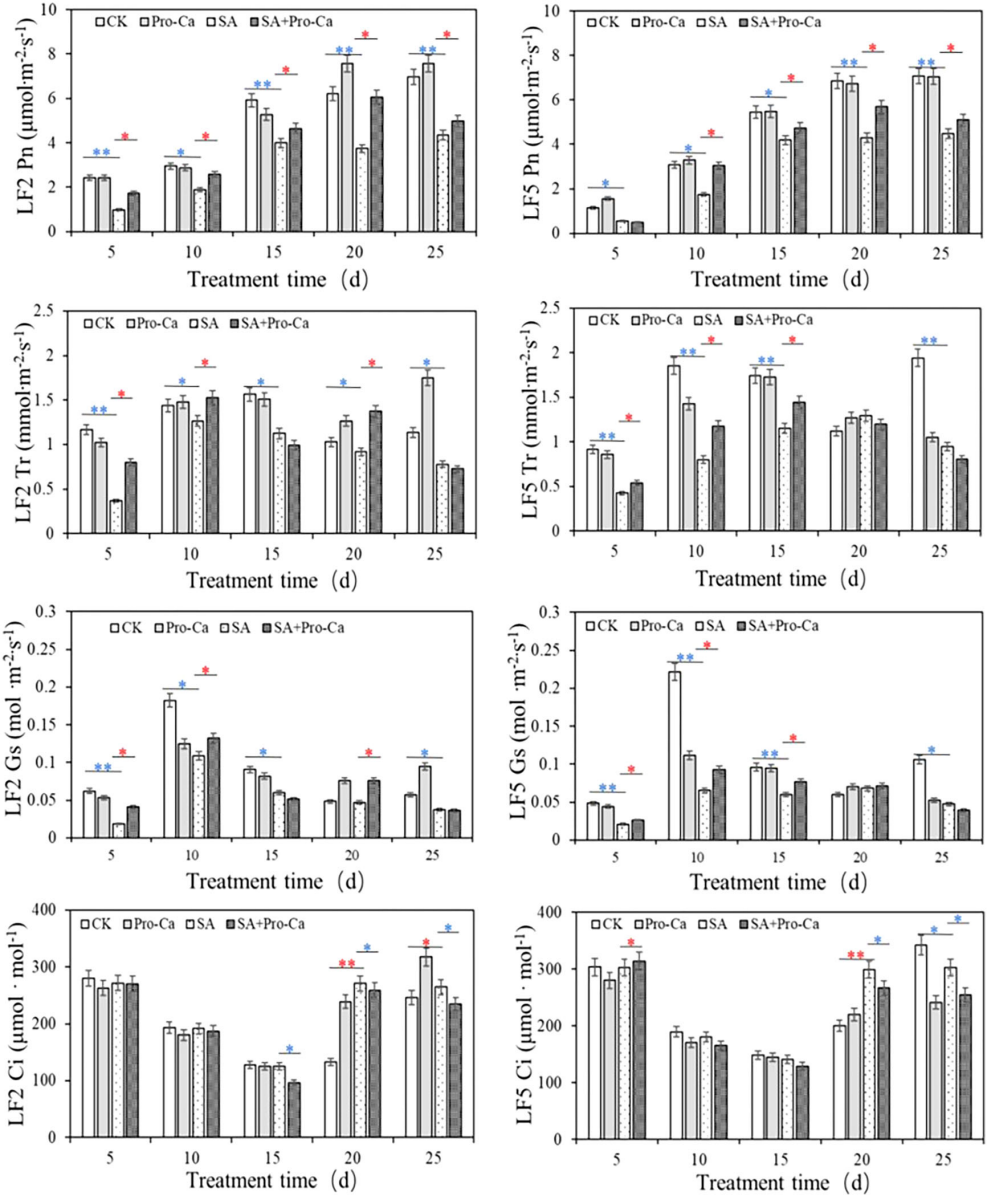
Effects of Pro-Ca on photosynthetic indexes of mung bean under saline-alkali stress.

Pro-Ca treatment positively affected photosynthesis in two mung bean varieties under saline-alkali stress (**Figure 4**). Compared with CK, SA stress significantly decreased the net photosynthetic rate (Pn), transpiration rate (Tr) and stomatal conductance (Gs), with reductions ranging from 32.64% to 70.54% across different time points. The application of Pro-Ca notably alleviated this inhibition. For instance, on day 20, Pn increased by 62.14% (LF2) and 32.45% (LF5) compared to the saline-alkali group. Tr and Gs also showed significant increases post-Pro-Ca treatment, by up to 53.68% and 53.98%, respectively. In addition, saline-alkali stress caused a significant increase in intercellular CO_2_ concentration (Ci) by day 20 (51.0% in LF2, 33.2% in LF5). Pro-Ca effectively reduced Ci by day 25. These findings indicate that Pro-Ca enhances photosynthetic performance by facilitating stomatal opening and improving gas exchange, thereby improving plant resilience to saline-alkali stress.

### 3.4 Pro-Ca increased osmotica content of mung bean under saline-alkali stress

**Figure 5:**
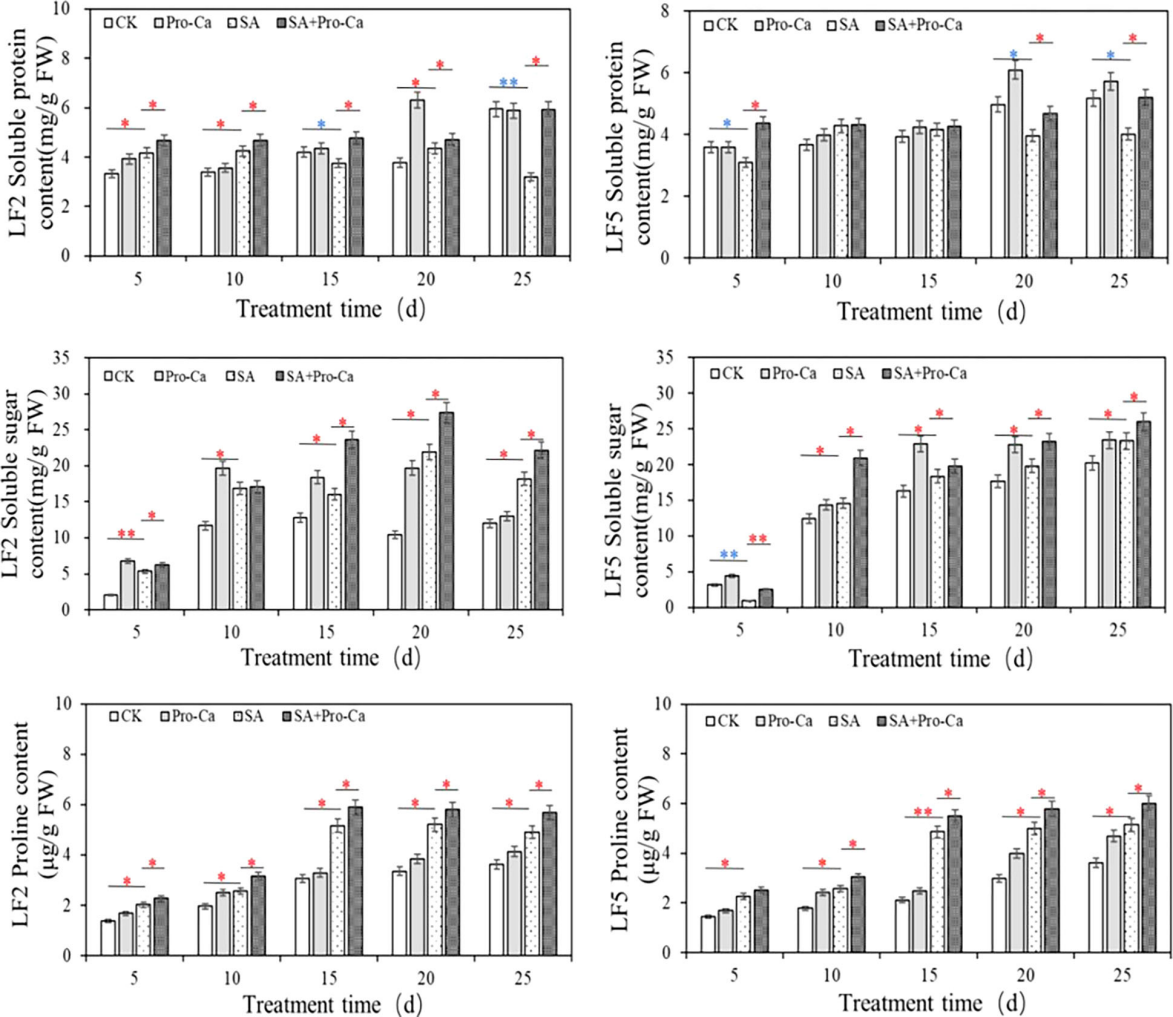
Effects of Pro-Ca on osmotica content under saline-alkali stress in mung bean.

The effects of Pro-Ca and saline-alkali stress on the osmotica content are shown in **Figure 5**. Under saline-alkali stress, the soluble protein content of LF2 leaves first increased and then decreased. At the 25th day, the soluble protein content decreased significantly by 46.10% and 22.45% in LF2 and LF5, respectively, compared to CK. Spraying Pro-Ca increased the soluble protein content to a certain extent, with significant increases of 96.25% and 30.83% at the 25th day in LF2 and LF5, respectively. The soluble sugar content in the leaves of the two mung bean varieties significantly increased under saline-alkali stress (except for the 5th day of LF5), and was further increased by spraying Pro-Ca, with an increase of 2.52%-49.37% and 10.00%-136.36% in LF2 and LF5, respectively. The proline content was significantly higher than that of the control at different times under saline-alkali stress, reaching a maximum increase at 15 days by 40.67% in LF2 and 56.47% in LF5, and then remained at a high level. With Pro-Ca spraying, the proline content further increased at all times by 12.20%-26.92% in LF2 and 11.22%-19.19% in LF5, compared with saline-alkali stress. These results indicate that soluble sugars and proline are important substances that increase osmotic regulation ability under saline-alkali stress in mung bean. The application of Pro-Ca improved osmotica content including soluble sugar, proline and soluble protein which enhanced the saline-alkali tolerance of the two mung bean varieties.

### 3.5 Pro-Ca alleviates peroxidation caused by saline-alkali stress in mung bean seedlings

**Figure 6:**
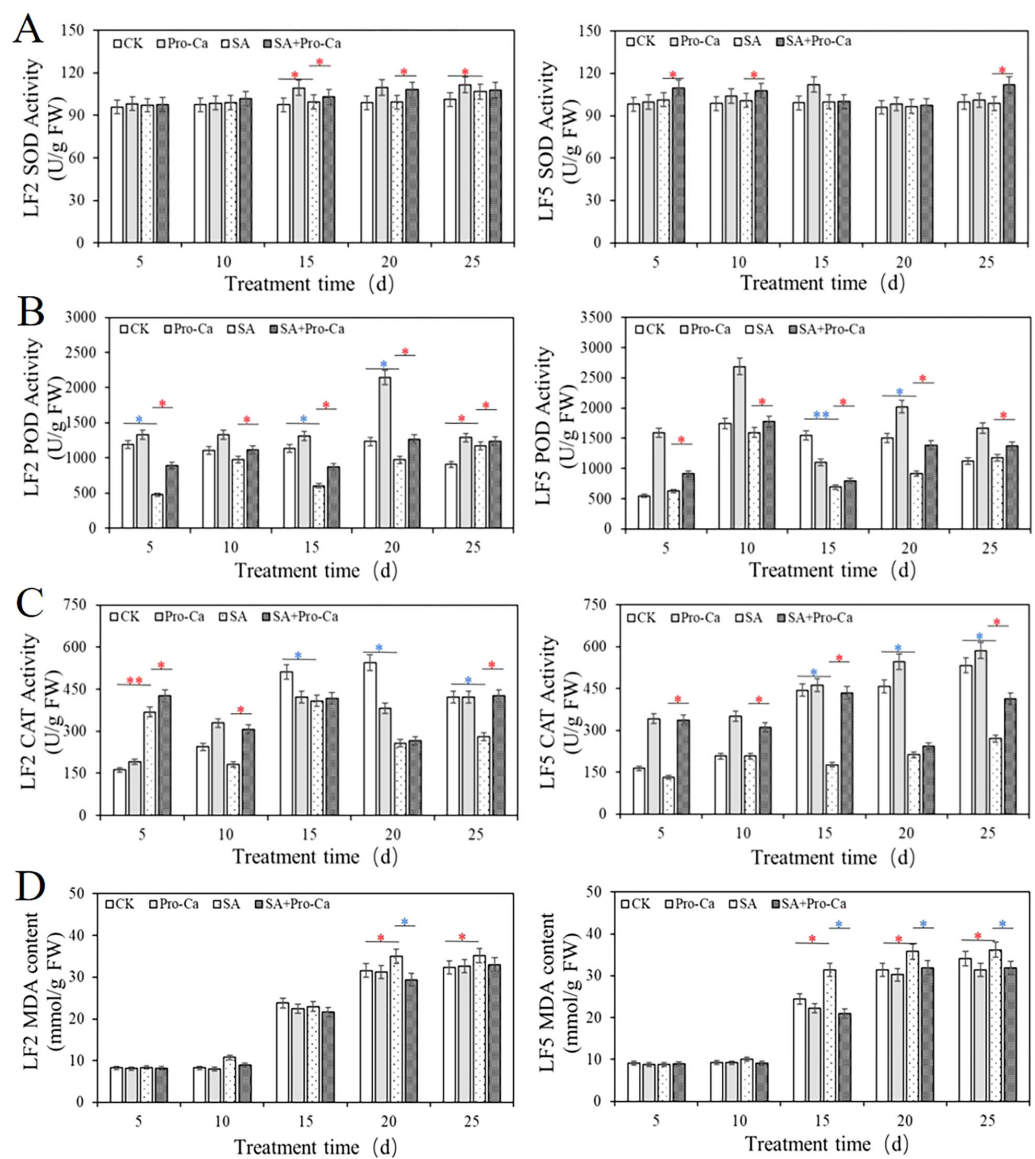
Effects of Pro-Ca on antioxidant enzyme activity and MDA content of mung bean under saline-alkali stress.

As shown in **Figure 6**, saline-alkali stress significantly altered antioxidant enzyme activities in two mung bean varieties. Superoxide dismutase (SOD) activity under saline-alkali stress increased significantly in LF2 on day 25 (5.30%) but remained unchanged in LF5. Pro-Ca application enhanced SOD activity in LF2 on days 15 and 20 (3.35%, 8.16%) and in LF5 on days 5, 10, and 25 (7.55% to 11.99%). Peroxidase (POD) activity decreased under saline-alkali stress, particularly 59.81% in LF2 on day 5 and 55.28% in LF5 on day 15. Pro-Ca treatment increased POD activity in both varieties across multiple time points, with increases ranging from 5.38% to 46.25%. Catalase (CAT) activity showed a decreased response in both varieties after day 15, declining from 25.45% to 60.00%. Pro-Ca restored CAT activity, leading to significant increases in both varieties, such as 40.58% increase in LF2 on day 10 and 60.53% increase in LF5 on day 5. Meanwhile, Pro-Ca reduced malondialdehyde (MDA) content, indicating alleviated oxidative damage, with significant reductions in LF2 (16.02%) and LF5 (10.75% to 33.19%). These results demonstrate that exogenous Pro-Ca enhances antioxidant defense and reduces membrane lipid peroxidation under saline-alkali stress.

### 3.6 The gene expression changes caused by saline-alkali and Pro-Ca in the roots are greater than those in the leaves

**Figure 7:**
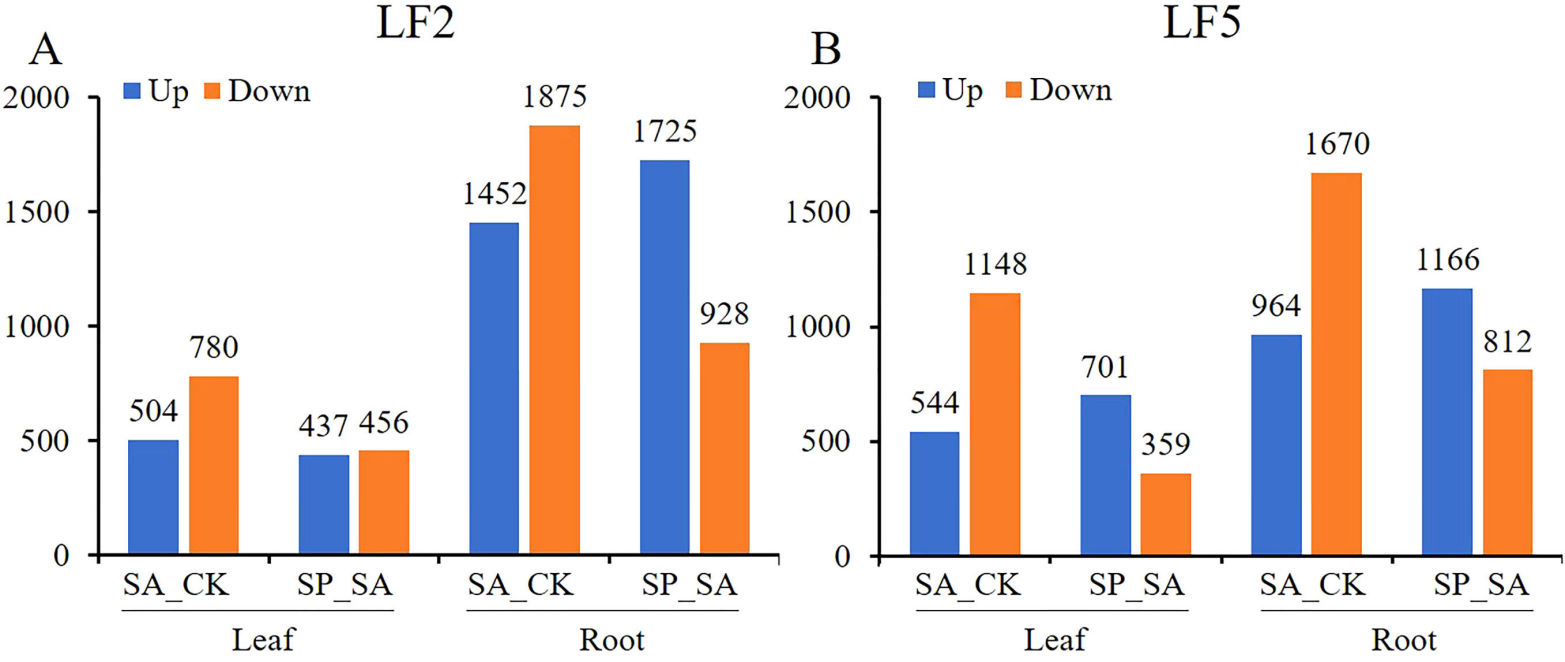
Statistical chart of the number of differentially expressed genes in the leaves and roots of LF2 and LF5. SA, SA group; CK, Control; SP, SA+Pro-Ca group.

As shown in **Figure 7**, Whether in the SA_CK group or the SP_SA group, the number of differentially expressed genes up- and downregulated in the roots of both varieties was higher than that in the leaves. The number of upregulated/downregulated genes in LF2 and LF5 leaves under saline-alkali stress was 504/780 and 544/1148, respectively, whereas in roots, it was 1452/1875 and 964/1670, respectively. With Pro-Ca spraying, the number of upregulated/downregulated genes in leaves was 437/456 and 701/359 in LF2 and LF5, respectively, while in roots, it was 1725/928 and 1166/812 in LF2 and LF5, respectively. This indicates that saline-alkali stress has a greater impact on gene expression in roots than in leaves, and Pro-Ca also has a more extensive effect on gene expression in roots to alleviate saline-alkali stress.

### 3.7 Pro-Ca enhanced calcium ion stability and Ca^2+^-dependent signals in roots to improve saline-alkali tolerance

Pro-Ca spraying upregulated the expression of multiple Ca^2+^ absorbance and release and calcium signal transmission proteins in the roots of both varieties, including calcium-transporting ATPase, calcium permeable stress-gated cation channel, calcium-binding protein, calmodulin, calmodulin-binding transcription activator, calcium-dependent protein kinase and so on (**Table 3**), indicating that Pro-Ca spraying may maintain calcium ion stability and promote the transmission of Ca^2+^ signals under saline-alkali stress. Moreover, 57 genes were upregulated in LF2, which is higher than LF5 (29 genes), suggesting that in the tolerant variety, Ca^2+^-dependent signals can be more effectively activated to enhance plant saline-alkali-tolerant ability.

**Table 3:** Differential expression of calcium ion stability and Ca^2+^-dependent signal protein genes in roots under saline-alkali stress by Pro-Ca spraying.

Varieties	Gene ID	Log2FoldChange	Annotation
LF2 (57 genes)	106773700/106766587/106766593/106777610/106768128	3.47/2.81/2.76/1.30/1.06	Calcium-transporting ATPase
	106775774/106768429	3.32/2.62	Calcium permeable stress-gated cation channel
	106760672/106755850/106761668/106763935/106752736/106778188/106767342/106768467/106757942/106777407/106761596/106772778/106758988/106780590/106754087/106752538/106762598/106759275/106761750	3.00/2.61/2.48/2.37/2.20/2.01/1.9/1.85/1.47/1.46/1.43/1.26/1.19/1.18/1.15/1.09/1.07/1.00/1.56	Calcium-binding protein
	106768268/106765775/106774059	2.68/2.31/1.12	Calmodulin-binding transcription activator
	106757185/106757518/106768263/111242324/106757360/106779964	2.18/2.12/2.01/1.92/1.42/1.41	Calmodulin-binding protein
	106775773/106764810/106755328	2.14/1.83/1.16	Calcium-dependent channel
	106770817/106767572/106767275/106771212/106758284/106771662	2.10/2.05/1.79/1.42/1.25/1.15	Calmodulin-like protein
	106757064/106754356/106777786/106757712/106769596	1.93/1.27/1.26/1.17/1.26	Calcium-dependent protein kinase
	106753788/106764777	1.82/1.25	Calcium-binding mitochondrial carrier protein
	106768878	1.70	Calcium uniporter protein 2
	106762074/106775813	1.60/1.39	Cation/calcium exchanger
	106758542/106775644	1.59/1.09	Calmodulin-regulated receptor-like kinase
	106767345	1.59	Calcium uptake protein
LF5 (29 genes)	106779443/106764412/106778188/106755850/106760672/106761668/106752736/106761596/106761750/106757942	5.94/4.24/2.22/2.19/2.17/2.15/1.62/1.20/1.05/1.01	Calcium-binding protein
	106766593/106766587/106773700	4.64/3.01/1.86	Calcium-transporting ATPase
	106771279/106757064/106757712	2.35/1.74/1.20	Calcium-dependent protein kinase
	106775773	2.34	Calcium-dependent channel
	106768429/106775774	2.29/1.42	Calcium permeable stress-gated cation channel
	106765775/106768268	2.19/1.75	Calmodulin-binding transcription activator
	106757518/111242324	2.17/1.13	Calmodulin binding protein-like
	106777218/106770817	1.77/1.36	Calmodulin-like protein
	106753788	1.32	Calcium-binding mitochondrial carrier protein
	106775006/106758542	1.16/1.01	Calmodulin-regulated receptor-like kinase
	106764699	1.14	Calcium uptake protein

### 3.8 The intensified metabolism of arginine generating NO and polyamines in roots increased saline-alkali tolerance of mung bean

**Figure 8:**
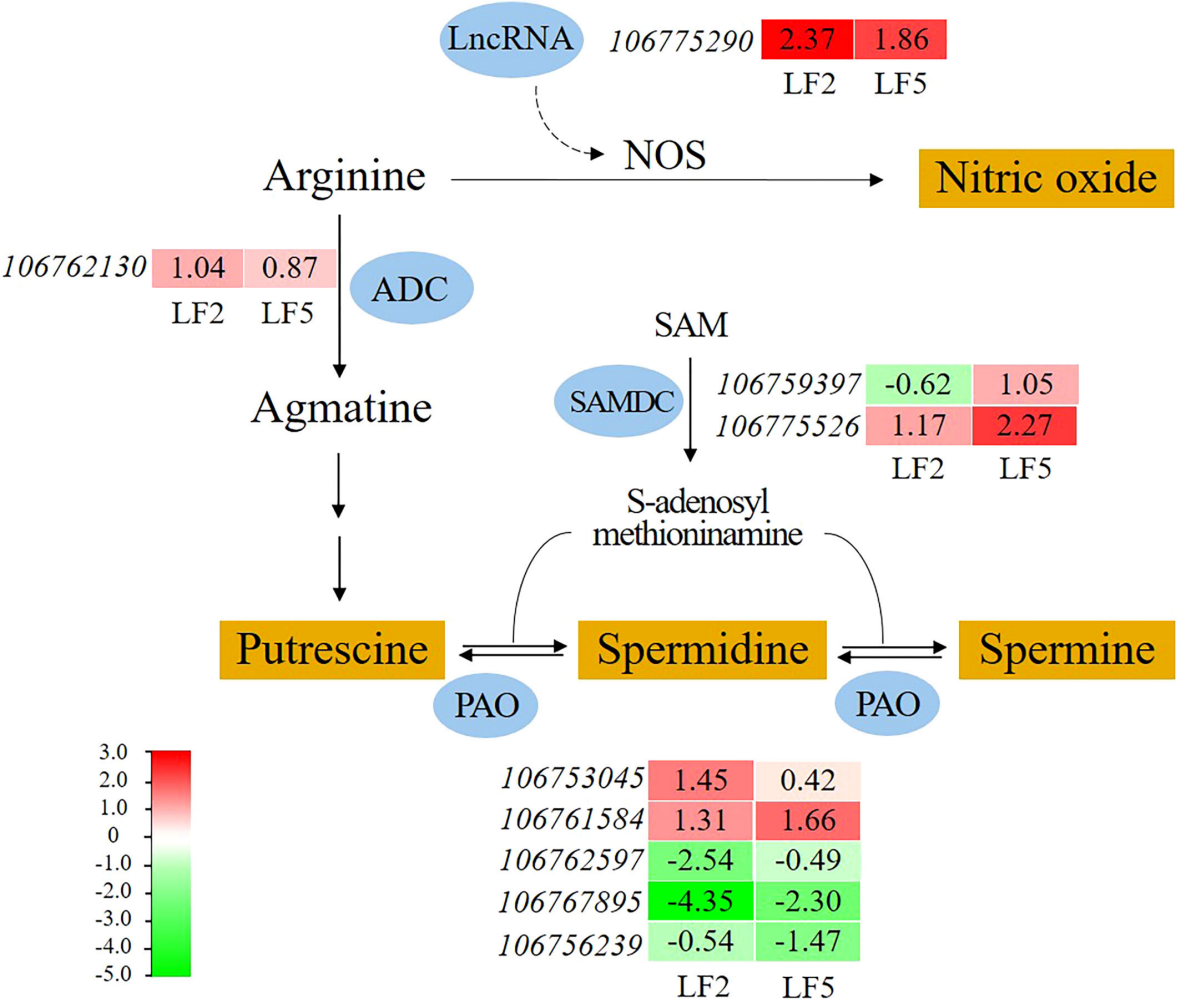
The effect of spraying Pro-Ca on arginine metabolism under saline-alkali stress. NOS, nitric oxide synthase; ADC, arginine decarboxylase; SAMDC, S-adenosylmethionine decarboxylase; PAO, polyamine oxidase. The red color in the heatmap indicates gene expression upregulation, while the green color indicates downregulation. The number represents the Log2FoldChange value of SA+Pro-Ca group compared to SA group.

Spraying Pro-Ca promoted arginine metabolism in the roots of both mung bean varieties (**Figure 8**). Nitric oxide synthase (NOS) is a key enzyme that catalyzes the synthesis of nitric oxide from arginine. Although we did not detect an increase in gene expression encoding NOS in this study, a lncRNA (106775290) in this pathway was found whose expression level upregulated by 2.37-fold and 1.86-fold in LF2 and LF5, respectively. The lncRNA may target NOS and affected NO synthesis. Moreover, the expression level of the arginine decarboxylase (ADC) gene (*106762130*) in LF2 was upregulated by 1.04-fold, which catalyzes the conversion of arginine to agmatine, and further putrescine. S-adenosylmethionine decarboxylase (SAMDC) gene (*106775526*) expression was upregulated by 1.17-fold and 2.27-flod in LF2 and LF5, respectively, contributing to the synthesis of spermidine and spermine. The gene expression of polyamine oxidase (PAO) which can catalyze the transition among putrescine, spermidine and spermine was significantly altered in both varieties of mung beans (*106753045*, *106761584*, *106762597*, *106767895*, *106756239*). These results suggest that polyamines also play an important role in alleviating saline-alkali stress in mung beans by Pro-Ca spraying.

### 3.9 Pro-Ca spraying strengthened saline-alkali tolerance by increasing the level of jasmonate/methyl-jasmonate in the roots

**Figure 9:**
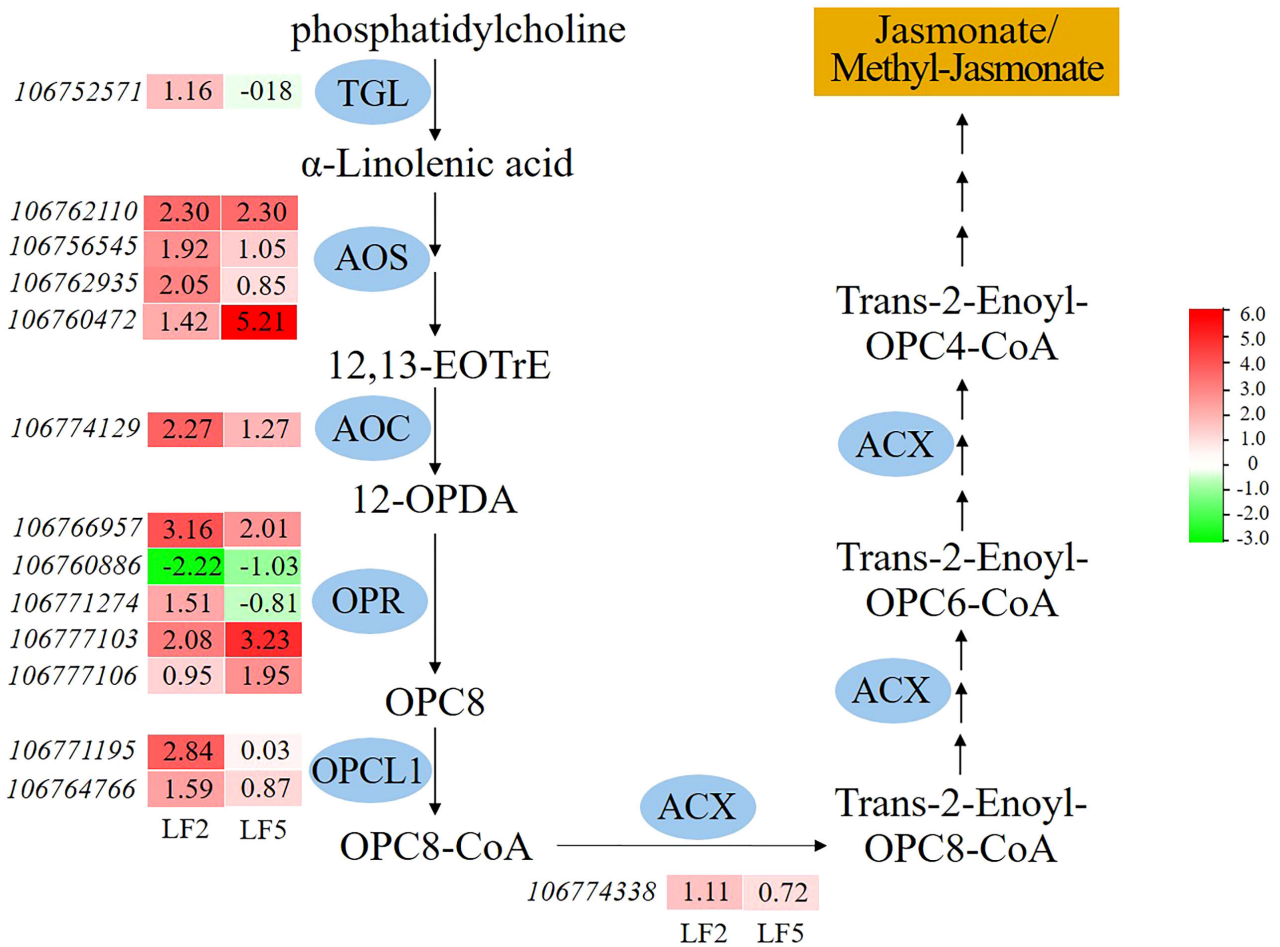
The effect of spraying Pro-Ca on alpha-Linolenic acid metabolism under saline-alkali stress. TGL, triacylglycerol lipase; AOS, allene oxide synthase; AOC, allene oxide cyclase; OPR, 12-oxophytodienoate reductase; OPCL1, 4-coumarate–CoA ligase; ACX, acyl-CoA. The red color in the heatmap indicates gene expression upregulation, while the green color indicates downregulation. The number represents the Log2FoldChange value of SA+Pro-Ca group compared to SA group.

In addition, spraying Pro-Ca improved the metabolism of α-linolenic acid in the roots, which is the substrate for the synthesis of jasmonate or methyl-jasmonate (**Figure 9**). In α-linolenic acid metabolism pathway, the gene expression levels of various enzymes including allene oxide synthase (AOS), allene oxide cyclase (AOC), 12-oxophytodienoate reductase (OPR) were significantly upregulated compared to saline-alkali control in both varieties. The enzymes of triacylglycerol lipase (TGL), 4-coumarate–CoA ligase (OPCL1), acyl-CoA oxidase (ACX) were significantly upregulated only in LF2. The results suggest that spraying Pro-Ca can enhance the tolerance to saline-alkali stress by increasing jasmonate/methyl-jasmonate synthesis of the roots, especially in LF2.

### 3.10 Pro-Ca spraying improved saline-alkali tolerance by altering abscisic acid signaling in the roots

Analysis of transcriptome data revealed that SA stress and spray of Pro-Ca did not significantly promote abscisic acid (ABA) synthesis (**Supplementary Table 3**), but enhanced the expression of key ABA signaling factors, particularly the protein phosphatase 2C gene (*106778050*) and the abscisic acid receptor PYL4 gene (*106764063*), which were significantly upregulated in LF2 (2.22-fold and 1.49-fold) and LF5 (1.13-fold and 2.21-fold) (**Table 4**). This suggests that the effect of spraying Pro-Ca on ABA signaling is mainly to enhance signal transduction rather than promote ABA synthesis.

**Table 4:** The differentially expressed genes of ABA signal key factors in roots under saline-alkali stress and Pro-Ca spraying.

Gene ID	Log2FoldChange				Annotation
	SP vs SA		SA vs CK		
	LF2	LF5	LF2	LF5	
106778050	2.22	1.13	0.91	-0.73	protein phosphatase 2C 51
106764063	1.49	2.21	0.06	-2.15	abscisic acid receptor PYL4
106770900	1.45	0.41	-1.40	-0.59	abscisic acid receptor PYL4-like
106758610	1.41	0.70	-0.68	-0.43	abscisic acid receptor PYL4
106755722	0.36	3.22	1.46	-0.93	abscisic acid receptor PYL4
106754671	1.15	0.49	0.07	-0.17	abscisic acid-insensitive 5-like protein 2

SP, SA+Pro-Ca group; SA, Saline-alkali stress; CK, Control.

## 4 Discussion

Saline-alkali stress is a major abiotic stress that impairs plant growth. Plant biomass serves as a reliable resistance indicator (Fang et al., 2021). Our results showed that saline-alkali stress reduced shoot and root dry weights in both mung bean varieties, with greater reductions in the sensitive LF5, particularly in root systems. Pro-Ca application significantly increased biomass in both varieties, consistent with the studies of Yu et al. (2025) and Feng et al. (2021) on soybean, demonstrating the role of Pro-Ca in enhancing crop stress adaptation. In agricultural practice, the combination of stress-tolerant varieties and Pro-Ca treatment will further improve the utilization of saline-alkali land.

Studies have shown that ultrastructural changes are closely related to biomass decline caused by saline-alkali stress (Geissler et al., 2009). In this experiment, saline-alkali stress severely damaged mung bean chloroplasts, causing swelling, rupture, and disorganized grana lamellae. These structural disruptions impaired photosynthetic function, leading to decreased Pn and chlorophyll content, ultimately inhibiting plant growth. The decline in photosynthetic capacity results from both stomatal limitation (due to partial stomatal closure) and non-stomatal limitation (due to reduced mesophyll cell activity) (Yi et al., 2018). Key photosynthetic parameters serve as critical indicators for distinguishing these factors. Generally, simultaneous decreases in Pn, Gs, and Ci point to stomatal limitation, whereas a decline in Pn and Gs alongside an increased in Ci suggests non-stomatal limitation (Gorbe and Calatayud, 2012). In this study, saline-alkali stress consistently significantly reduced Pn and Gs in LF2 and LF5, while Ci increased until day 20. These results indicate that photosynthetic limitation was primarily governed by non-stomatal factors.

Exogenous Pro-Ca application significantly increased Pn under saline-alkali stress, demonstrating effective alleviation of photosynthetic limitation. However, this alleviation was not mediated by stomatal factors. This conclusion is supported by two key findings: Firstly, the observed Pn increase was accompanied by a significant Ci decrease in later stages, excluding stomatal limitation as the primary cause; Secondly, transcriptome analysis of LF5 revealed that saline-alkali stress downregulated all 57 DEGs in photosynthesis-related pathways (including photosynthesis-antenna proteins, photosynthesis, and carbon fixation), while Pro-Ca treatment upregulated all 33 DEGs in these same pathways (**Supplementary Table 4**). These results clearly indicate that Pro-Ca ameliorates photosynthetic limitation by specifically enhancing the expression of photosynthetic proteins rather than through stomatal regulation.

Saline-alkali stress induces cellular water loss, leading to physiological drought and ROS accumulation that disrupts cellular metabolism and triggers membrane lipid peroxidation (Fang et al., 2021). Plants counteract these effects through coordinated osmotic regulation and antioxidant defense mechanisms (Fang et al., 2021; Hou et al., 2023; Jia et al., 2019). Our observations revealed mitochondrial reduce in stressed tissues, particularly in LF5, impairing ROS scavenging capacity. Pro-Ca application partially restored mitochondrial numbers, elevated antioxidant enzyme activities (SOD, POD, CAT), and reduced oxidative damage, aligning with established correlations between enhanced antioxidant activity and improved abiotic stress tolerance (Chen et al., 2024, 2023; Ma et al., 2024). Concurrently, Pro-Ca promoted osmotic adjustment through accumulating key osmolytes (proline, soluble sugars, proteins), a critical mechanism for maintaining cellular water potential and facilitating stress adaptation (Singh et al., 2022). Proline functions dually as an osmoprotectant and signaling molecule, regulating gene expression and pH stability (Alvarez et al., 2022). Soluble proteins serve as indicators of plant metabolic activity and antioxidant capacity (Zhang et al., 2021). Soluble sugars act as both carbon sources and osmotic stress signals that interact with phytohormones (e.g., ABA and ethylene) to form an integrated stress response network (Kaur et al., 2021). These coordinated mechanisms demonstrate how Pro-Ca enhances plant tolerance to saline-alkali stress through simultaneous modulation of antioxidant capacity and osmotic homeostasis.

**Figure 10:**
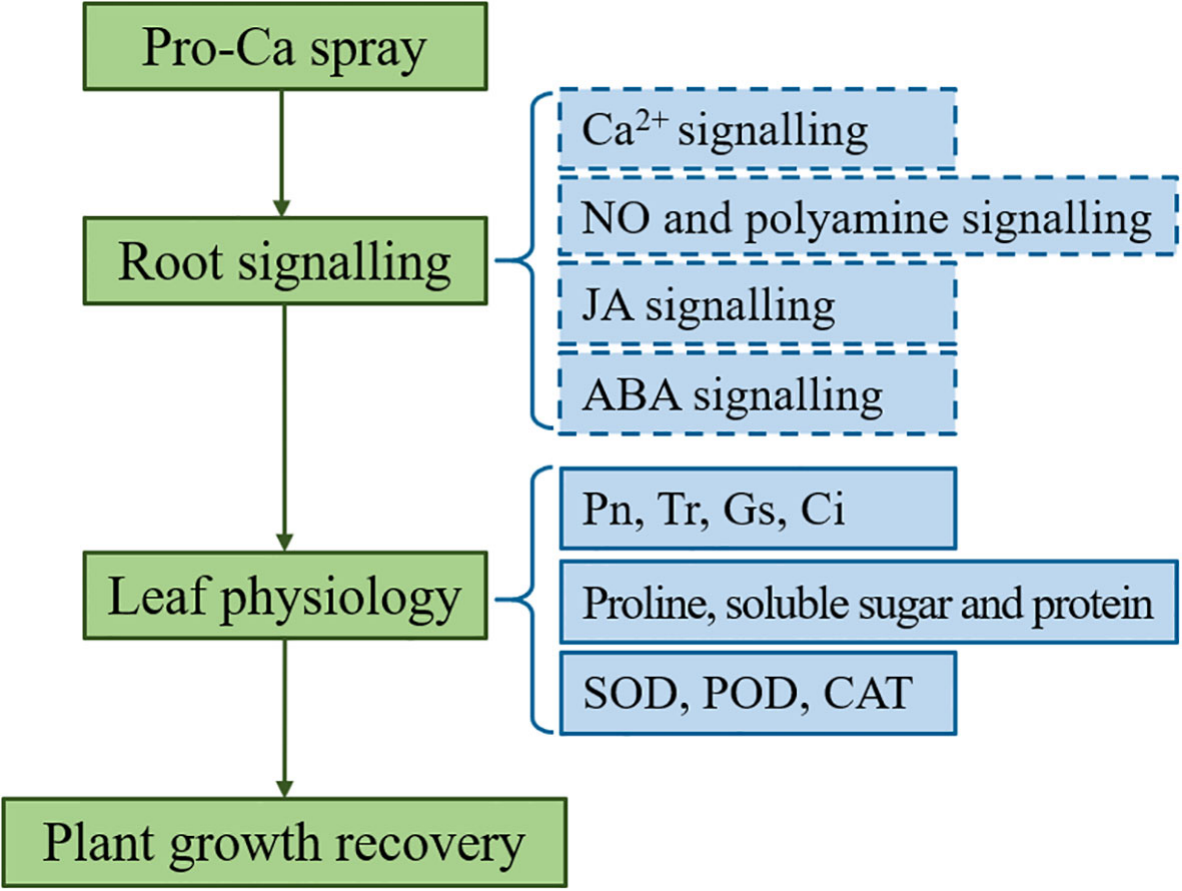
Mechanism analysis of spraying Pro-Ca to restore plant growth under Saline-alkali stress. The dashed line represents the inferred content based on transcriptome data, which requires further experimental verification.

The response of plants to abiotic stress is closely related to multiple signals (Waadt et al., 2022; Aslam et al., 2021). Many studies have reported the positive effects of Ca^2+^, NO, polyamine, JA and ABA signals on photosynthesis (Bai et al., 2022), antioxidant activity (Lang et al., 2020), and osmotic regulation (Choudhary et al., 2023) in plant responses to salt alkali stress. The regulation of Pro-Ca on antioxidant and osmotic systems appears to be mediated through its promotion of Ca^2+^ uptake, transport, and signaling under saline-alkali stress (**Table 3**). The underlying mechanism involves activation of Ca^2+^ channels that facilitate extracellular Ca^2+^ influx, with Calcineurin B-like proteins (CBLs) serving as sensor relays that transmit Ca^2+^ signals through conformational changes, and Calcium-dependent protein kinases (CDPKs) functioning as sensor-responders that convey these signals into physiological responses via substrate phosphorylation (Boudsocq and Laurière, 2005; Marcec et al., 2019; Yip Delormel and Boudsocq, 2019). This calcium signaling cascade stimulates both the synthesis of osmotic regulators and the activation of antioxidant enzymes, which collectively scavenge ROS and protect cellular membranes (Chinnusamy et al., 2004). NO serves as a crucial signaling molecule in plant environmental adaptation (Gupta et al., 2011). Our investigation revealed upregulated lncRNA associated with NO biosynthesis via the arginine metabolic pathway, suggesting its potential regulatory role in NO-mediated stress responses. LncRNAs have been reviewed owning to functional diversity both cis and trans to alter targeted DNA, RNA and proteins via epigenetic modifications and direct molecular interactions (Kaikkonen et al., 2011; Lee, 2012). Miao et al. (2018) reported that lncRNA LEENE transcriptionally activates endothelial NO synthase (eNOS) to promote vascular function. Bernardi et al. (2025) revealed that lncRNA DSCR9 post-translationally regulates eNOS activity by reducing its phosphorylation under high pressure conditions without altering total protein levels. Wang et al. (2021) reported that lncRNA LINC00973 enhances lactate dehydrogenase A (LDHA) activity through direct binding. These findings collectively demonstrate lncRNAs’ capacity to fine-tune cellular processes at multiple regulatory levels. Interestingly, under saline-alkali stress with Pro-Ca treatment, high lncRNA expression didn’t increase NOS levels, suggesting their role in NO synthesis may involve enzyme activity modulation rather than expression changes. According to the results of this experiment, we have summarized a concise Pro-Ca action model under saline-alkali stress (**Figure 10**), which indicates that Pro-Ca treatment stimulates root signal transduction including Ca^2+^, NO, polyamine, JA and ABA. The alterations in these signals have led to physiological changes in leaves and ultimately resulting in plant growth recovery. Unfortunately, we did not measure the intensity of these signals, including Ca^2+^ concentration, contents of NO, polyamines, JA and ABA, and related enzyme activity. Therefore, these speculated mechanisms require further experimental verification.

## 5 Conclusion

Exogenous Pro-Ca application significantly enhanced mung bean growth under saline-alkali stress, protected the integrity of subcellular structures, enhanced the photosynthetic ability, increased osmotic regulator content, and relieved membrane lipid peroxidation. These changes may be related to calcium signal transmission and NO, polyamines and jasmonic acid synthesis in root tissues under saline-alkali stress. Our results provide novel insights into the physiological and molecular mechanisms underlying Pro-Ca-induced saline-alkali tolerance in mung bean seedlings. Furthermore, these findings reveal the complex action of exogenous Pro-Ca and warrant further research into its stress-mitigating mechanisms.

## Data Availability

The datasets presented in this study can be found in online repositories. The names of the repository/repositories and accession number(s) can be found in the article/**Supplementary Material**.
