# Supplementary material for: Foliar spray of prohexadione-calcium improves the adaptability of mung bean to saline-alkali stress
Source: Front Plant Sci. 2025 Oct 24;16:1681992. doi: 10.3389/fpls.2025.1681992 (PMC12592081; doi:10.3389/fpls.2025.1681992)
Supplement: Supplementary file 1 [file Table1.docx]

**Supplementary Table 1 Reference genome alignment and analysis**

| **Samples** | **Clean Reads(M)** | **Total mapped%** | **Unique mapped%** | **Q20** |
| --- | --- | --- | --- | --- |
| LF2-L_CK-1 | 45694212 | 91.33 | 87.65 | 98.24 |
| LF2-L_CK-2 | 45029918 | 92.42 | 89.11 | 97.89 |
| LF2-L_CK-3 | 47811200 | 91.62 | 88.15 | 97.94 |
| LF2-L_SA-1 | 52504526 | 91.04 | 86.79 | 97.91 |
| LF2-L_SA-2 | 48297254 | 92.57 | 89.19 | 98.32 |
| LF2-L_SA-3 | 46302090 | 91.03 | 87.23 | 97.95 |
| LF2-L_SA_Pro-Ca-1 | 54161366 | 92.86 | 88.16 | 98.50 |
| LF2-L_SA_Pro-Ca-2 | 54014644 | 93.40 | 87.56 | 98.92 |
| LF2-L_SA_Pro-Ca-3 | 46853964 | 92.38 | 88.31 | 98.20 |
| LF2-R_CK-1 | 48946216 | 92.49 | 90.37 | 98.39 |
| LF2-R_CK-2 | 45402054 | 91.76 | 89.75 | 98.39 |
| LF2-R_CK-3 | 46440674 | 92.69 | 90.66 | 98.42 |
| LF2-R_SA-1 | 46759150 | 89.80 | 87.83 | 98.41 |
| LF2-R_SA-2 | 45952538 | 87.91 | 86.06 | 98.42 |
| LF2-R_SA-3 | 49436738 | 88.09 | 86.15 | 98.11 |
| LF2-R_SA_Pro-Ca-1 | 48215610 | 89.77 | 87.92 | 98.13 |
| LF2-R_SA_Pro-Ca-2 | 44881032 | 90.74 | 88.84 | 98.11 |
| LF2-R_SA_Pro-Ca-3 | 46560820 | 89.98 | 88.07 | 98.12 |
| LF5-L_CK-1 | 46015930 | 92.62 | 87.54 | 98.87 |
| LF5-L_CK-2 | 46097728 | 91.06 | 85.89 | 98.50 |
| LF5-L_CK-3 | 46107782 | 91.90 | 87.23 | 98.15 |
| LF5-L_SA-1 | 48042222 | 91.71 | 87.88 | 98.20 |
| LF5-L_SA-2 | 48686126 | 92.95 | 88.89 | 98.20 |
| LF5-L_SA-3 | 50330432 | 92.95 | 89.67 | 98.22 |
| LF5-L_SA_Pro-Ca-1 | 45792456 | 95.73 | 88.54 | 98.20 |
| LF5-L_SA_Pro-Ca-2 | 48536766 | 91.77 | 87.56 | 98.45 |
| LF5-L_SA_Pro-Ca-2 | 48125066 | 92.78 | 88.45 | 98.46 |
| LF5-R_CK-1 | 48310306 | 92.02 | 89.93 | 98.09 |
| LF5-R_CK-2 | 48839062 | 91.41 | 89.43 | 98.09 |
| LF5-R_CK-3 | 48066304 | 90.16 | 88.20 | 98.13 |
| LF5-R_SA-1 | 45333092 | 90.75 | 88.81 | 98.20 |
| LF5-R_SA-2 | 46690600 | 91.68 | 89.69 | 98.06 |
| LF5-R_SA-3 | 48339152 | 90.74 | 88.74 | 98.16 |
| LF5-R_SA_Pro-Ca-1 | 44719678 | 91.84 | 90.12 | 98.46 |
| LF5-R_SA_Pro-Ca-2 | 39746080 | 91.11 | 89.12 | 98.08 |
| LF5-R_SA_Pro-Ca-2 | 49527338 | 90.26 | 88.30 | 98.18 |
