# Supplementary material for: Foliar spray of prohexadione-calcium improves the adaptability of mung bean to saline-alkali stress
Source: Front Plant Sci. 2025 Oct 24;16:1681992. doi: 10.3389/fpls.2025.1681992 (PMC12592081; doi:10.3389/fpls.2025.1681992)
Supplement: Supplementary file 2 [file Table2.docx]

Supplementary table 2 GO and KEGG enrichment by Pro-Ca spraying under SA stress

| **Groups** | **GO** | | | **KEGG** | | |
| --- | --- | --- | --- | --- | --- | --- |
|  | Description | Padj | Count | Description | Padj | Count |
| L2 Root | cell wall | 2.21E-05 | 12 | Plant-pathogen interaction | 8.92E-11 | 47 |
|  | external encapsulating structure | 2.21E-05 | 12 | Plant hormone signal transduction | 0.0001 | 44 |
|  | apoplast | 2.21E-05 | 12 | alpha-Linolenic acid metabolism | 0.0191 | 13 |
|  | extracellular region | 4.45E-05 | 14 | Arginine and proline metabolism | 0.0191 | 13 |
|  | cell periphery | 0.0295 | 17 | MAPK signaling pathway - plant | 0.0191 | 24 |
|  | DNA-binding TF activity | 4.53E-08 | 94 | Phenylpropanoid biosynthesis | 0.0308 | 24 |
|  | transcription regulator activity | 4.53E-08 | 97 | Taurine and hypotaurine metabolism | 0.0425 | 5 |
|  | xyloglucan:xyloglucosyl transferase activity | 0.0014 | 12 |  |  |  |
|  | tetrapyrrole binding | 0.0030 | 68 |  |  |  |
|  | heme binding | 0.0037 | 67 |  |  |  |
|  | ADP binding | 0.0037 | 53 |  |  |  |
|  | oxidoreductase activity, acting on paired donors, with incorporation or reduction of O_2_ | 0.0051 | 52 |  |  |  |
|  | calcium ion binding | 0.0199 | 33 |  |  |  |
|  | monooxygenase activity | 0.0255 | 10 |  |  |  |
|  | lipase activity | 0.0255 | 6 |  |  |  |
|  | sequence-specific DNA binding | 0.0353 | 37 |  |  |  |
|  | peroxidase activity | 0.0469 | 25 |  |  |  |
| L5 Root | extracellular region | 0.0324 | 9 | Phenylpropanoid biosynthesis | 0.031 | 14 |
|  | cell wall | 0.0324 | 7 | Carotenoid biosynthesis | 0.035 | 7 |
|  | external encapsulating structure | 0.0324 | 7 |  |  |  |
|  | apoplast | 0.0324 | 7 |  |  |  |
|  | heme binding | 4.21E-05 | 62 |  |  |  |
|  | tetrapyrrole binding | 4.21E-05 | 62 |  |  |  |
|  | DNA-binding TF activity | 0.0001 | 68 |  |  |  |
|  | transcription regulator activity | 0.0003 | 69 |  |  |  |
|  | endopeptidase inhibitor activity | 0.0003 | 14 |  |  |  |
|  | peptidase inhibitor activity | 0.0003 | 14 |  |  |  |
|  | peptidase regulator activity | 0.0003 | 14 |  |  |  |
|  | endopeptidase regulator activity | 0.0003 | 14 |  |  |  |
|  | oxidoreductase activity, acting on paired donors, with incorporation or reduction of O_2_ | 0.0020 | 44 |  |  |  |
|  | iron ion binding | 0.0020 | 44 |  |  |  |
|  | enzyme inhibitor activity | 0.0021 | 25 |  |  |  |
|  | peroxidase activity | 0.0097 | 22 |  |  |  |
|  | oxidoreductase activity, acting on peroxide as acceptor | 0.0114 | 22 |  |  |  |
|  | antioxidant activity | 0.0212 | 22 |  |  |  |
|  | transferase activity, transferring glycosyl groups | 0.0227 | 53 |  |  |  |
|  | transferase activity, transferring hexosyl groups | 0.0227 | 45 |  |  |  |
|  | sequence-specific DNA binding | 0.0323 | 30 |  |  |  |
|  | transferase activity, transferring acyl groups other than amino-acyl groups | 0.04205 | 21 |  |  |  |
| L2 Leaf | ADP binding | 6.84E-06 | 33 | Plant-pathogen interaction | 0.0007 | 19 |
|  | pattern binding | 0.0494 | 9 | MAPK signaling pathway - plant | 0.0122 | 14 |
|  | polysaccharide binding | 0.0494 | 9 | Flavonoid biosynthesis | 0.0393 | 7 |
| L5 Leaf | polysaccharide metabolic process | 0.0482 | 15 | Photosynthesis-antenna proteins | 2.60E-16 | 16 |
|  | cellular glucan metabolic process | 0.0482 | 13 | Circadian rhythm - plant | 3.12E-08 | 17 |
|  | glucan metabolic process | 0.0482 | 13 | Carotenoid biosynthesis | 0.0011 | 10 |
|  | cellular polysaccharide metabolic process | 0.0482 | 13 | Tropane, piperidine and pyridine alkaloid biosynthesis | 0.0137 | 9 |
|  | cell wall | 0.0042 | 7 | Starch and sucrose metabolism | 0.0137 | 20 |
|  | external encapsulating structure | 0.0042 | 7 | Porphyrin metabolism | 0.019417 | 9 |
|  | apoplast | 0.0042 | 7 | Carbon fixation in photosynthetic organisms | 0.039394 | 10 |
|  | extracellular region | 0.0083 | 8 |  |  |  |
|  | oxidoreductase activity, acting on paired donors, with incorporation or reduction of O_2_ | 4.58E-07 | 38 |  |  |  |
|  | iron ion binding | 1.34E-06 | 37 |  |  |  |
|  | heme binding | 5.34E-05 | 38 |  |  |  |
|  | tetrapyrrole binding | 5.34E-05 | 38 |  |  |  |
|  | transferase activity, transferring hexosyl groups | 0.0045 | 33 |  |  |  |
|  | glucosyltransferase activity | 0.0064 | 14 |  |  |  |
|  | hydrolase activity, acting on glycosyl bonds | 0.0082 | 30 |  |  |  |
|  | lyase activity | 0.0091 | 18 |  |  |  |
|  | enzyme inhibitor activity | 0.0101 | 15 |  |  |  |
|  | microtubule motor activity | 0.0101 | 11 |  |  |  |
|  | xyloglucan:xyloglucosyl transferase activity | 0.0122 | 7 |  |  |  |
|  | cysteine-type endopeptidase inhibitor activity | 0.0187 | 4 |  |  |  |
|  | hydrolase activity, hydrolyzing O-glycosyl compounds | 0.0187 | 27 |  |  |  |
|  | microtubule binding | 0.0215 | 11 |  |  |  |
|  | oxidoreductase activity, acting on single donors with incorporation of O_2_ | 0.0291 | 8 |  |  |  |
|  | motor activity | 0.0353 | 11 |  |  |  |
|  | tubulin binding | 0.0361 | 11 |  |  |  |
|  | transferase activity, transferring glycosyl groups | 0.0361 | 33 |  |  |  |
|  | oxidoreductase activity, acting on the aldehyde or oxo group of donors, NAD/NADP as acceptor | 0.0361 | 4 |  |  |  |
|  | oxidoreductase activity, acting on the aldehyde or oxo group of donors | 0.0378 | 5 |  |  |  |
|  | endopeptidase inhibitor activity | 0.0378 | 7 |  |  |  |
|  | peptidase inhibitor activity | 0.0378 | 7 |  |  |  |
|  | peptidase regulator activity | 0.0378 | 7 |  |  |  |
|  | endopeptidase regulator activity | 0.0378 | 7 |  |  |  |
|  | FAD binding | 0.0483 | 4 |  |  |  |
