# Supplementary material for: Foliar spray of prohexadione-calcium improves the adaptability of mung bean to saline-alkali stress
Source: Front Plant Sci. 2025 Oct 24;16:1681992. doi: 10.3389/fpls.2025.1681992 (PMC12592081; doi:10.3389/fpls.2025.1681992)
Supplement: Supplementary file 3 [file Table3.docx]

Supplementary Table 3 The differentially expressed genes of ABA biosynthesis in roots under saline-alkali stress and pro-Ca spraying

| Gene ID | Log2FoldChange | | | | | Annotation |
| --- | --- | --- | --- | --- | --- | --- |
|  | SP-SA | |  | SA-CK | |  |
|  | LF2 | LF5 |  | LF2 | LF5 |  |
| 106763718 | -2.11 | -2.62 |  | 2.92 | 0.38 | beta-carotene hydroxylase 2 |
| 106768400 | 0.95 | 2.66 |  | 3.59 | -0.09 | zeaxanthin epoxidase |
| 106755472 | 2.97 | 2.79 |  | -1.04 | -3.17 | abscisic acid 8'-hydroxylase 1 |
| 106758055 | -0.59 | 0.21 |  | -1.17 | -1.56 | protein LUTEIN DEFICIENT 5 |
| 106765025 | -1.09 | -1.35 |  | 1.00 | -0.21 | phytoene synthase 2 |
| 106770994 | -0.20 | -1.27 |  | 0.47 | 0.80 | beta-carotene hydroxylase 2 |
