# Supplementary material for: Foliar spray of prohexadione-calcium improves the adaptability of mung bean to saline-alkali stress
Source: Front Plant Sci. 2025 Oct 24;16:1681992. doi: 10.3389/fpls.2025.1681992 (PMC12592081; doi:10.3389/fpls.2025.1681992)
Supplement: Supplementary file 4 [file Table4.docx]

Supplementary Table 4 Photosynthetic-related pathways using KEGG database and differentially expressed genes by pro-Ca spraying under saline-alkali stress in LF5.

|  | **SA_CK** | | | | **SP_SA** | | | |
| --- | --- | --- | --- | --- | --- | --- | --- | --- |
| **Pathways** | **P value** | **Gene ID** | **Log2FoldChange** | **Annotation** | **P value** | **Gene ID** | **Log2FoldChange** | **Annotation** |
| Photosynthesis-antenna proteins | 7.76e-16 | *106774390* | -5.49 | Chlorophyll a-b binding protein of LHCII type 1-like | 2.58E-18 | *106752877* | 4.06 | Chlorophyll a-b binding protein 3 |
|  | (16) | *106777957* | -5.28 | Chlorophyll a-b binding protein of LHCII type 1-like | (16) | *106765086* | 3.60 | Chlorophyll a-b binding protein 215 |
|  |  | *106752877* | -4.70 | Chlorophyll a-b binding protein 3 |  | *106774260* | 2.4 | Chlorophyll a-b binding protein CP29.1 |
|  |  | *106765086* | -4.35 | Chlorophyll a-b binding protein 215 |  | *106774390* | 3.75 | Chlorophyll a-b binding protein of LHCII type 1-like |
|  |  | *106756425* | -3.84 | Chlorophyll a-b binding protein 13 |  | *106765279* | 2.64 | Chlorophyll a-b binding protein 8 |
|  |  | *106765279* | -3.43 | Chlorophyll a-b binding protein 8 |  | *106777957* | 3.70 | Chlorophyll a-b binding protein of LHCII type 1-like |
|  |  | *106774260* | -3.26 | Chlorophyll a-b binding protein CP29.1 |  | *106756707* | 2.68 | Chlorophyll a-b binding protein P4 |
|  |  | *106756707* | -3.16 | Chlorophyll a-b binding protein P4 |  | *106778184* | 1.78 | Chlorophyll a-b binding protein |
|  |  | *106780309* | -2.86 | Chlorophyll a-b binding protein 13 |  | *106775787* | 1.68 | Chlorophyll a-b binding protein CP24 10A |
|  |  | *106775787* | -2.81 | Chlorophyll a-b binding protein CP24 10A |  | *106760190* | 1.49 | Chlorophyll a-b binding protein |
|  |  | *106778184* | -2.73 | Chlorophyll a-b binding protein |  | *106756425* | 3.07 | Chlorophyll a-b binding protein 13 |
|  |  | *106775014* | -2.23 | Chlorophyll a-b binding protein 6A |  | *106780309* | 1.88 | Chlorophyll a-b binding protein 13 |
|  |  | *106774152* | -2.21 | Chlorophyll a-b binding protein CP24 10A |  | *106772865* | 1.42 | Chlorophyll a-b binding protein CP26 |
|  |  | *106772865* | -2.10 | Chlorophyll a-b binding protein CP26 |  | *106775014* | 1.50 | Chlorophyll a-b binding protein 6A |
|  |  | *106760190* | -1.89 | Chlorophyll a-b binding protein |  | *106758023* | 1.35 | Chlorophyll a-b binding protein CP29.3 |
|  |  | *106758023* | -1.88 | Chlorophyll a-b binding protein CP29.3 |  | *106774152* | 1.16 | Chlorophyll a-b binding protein CP24 10A |
| Photosynthesis | 6.38e-11 | *106775678* | -4.47 | Ferredoxin-like | 0.013 | *106759760* | 1.65 | Photosystem II 22 kDa protein |
|  | (20) | *106758006* | -3.58 | Photosystem II reaction center PSB28 protein | (7) | *106760980* | 1.57 | Photosystem I reaction center subunit psaK |
|  |  | *106775681* | -3.57 | Ferredoxin-A |  | *106775681* | 1.72 | Ferredoxin-A |
|  |  | *106763549* | -2.85 | Photosystem I reaction center subunit N |  | *106763549* | 1.70 | Photosystem I reaction center subunit N |
|  |  | *106767622* | -2.09 | Photosystem II core complex proteins psbY |  | *106758006* | 2.61 | Photosystem II reaction center PSB28 protein |
|  |  | *106759760* | -2.01 | Photosystem II 22 kDa protein |  | *106754761* | 1.06 | Photosystem I subunit O |
|  |  | *106760980* | -2.00 | Photosystem I reaction center subunit psaK |  | *106767622* | 1.03 | Photosystem II core complex proteins psbY |
|  |  | *106754761* | -1.83 | Photosystem I subunit O |  |  |  |  |
|  |  | *106765722* | -1.76 | Photosystem I reaction center subunit III |  |  |  |  |
|  |  | *106775680* | -1.65 | Ferredoxin-like |  |  |  |  |
|  |  | *106768113* | -1.53 | Oxygen-evolving enhancer protein 1 |  |  |  |  |
|  |  | *106761826* | -1.47 | Photosystem II 10 kDa polypeptide |  |  |  |  |
|  |  | *106775679* | -1.44 | Ferredoxin-like |  |  |  |  |
|  |  | *106778792* | -1.42 | Oxygen-evolving enhancer protein 2 |  |  |  |  |
|  |  | *106764695* | -1.28 | Oxygen-evolving enhancer protein 2 |  |  |  |  |
|  |  | *106776372* | -1.25 | Photosystem I reaction center subunit IV A |  |  |  |  |
|  |  | *106769516* | -1.24 | Ferredoxin--NADP reductase |  |  |  |  |
|  |  | *106769759* | -1.15 | Photosystem I reaction center subunit IV |  |  |  |  |
|  |  | *106778062* | -1.11 | Oxygen-evolving enhancer protein 1 |  |  |  |  |
|  |  | *106773542* | -1.07 | Photosystem I reaction center subunit VI-2 |  |  |  |  |
| Carbon fixation in photosynthetic organisms | 7.29e-09 | *106760805* | -3.66 | Phosphoenolpyruvate carboxylase | 0.0027 | *106752764* | 1.08 | Glyceraldehyde-3-phosphate dehydrogenase |
|  | (21) | *106778590* | -2.74 | Phosphoenolpyruvate carboxylase 2 | (10) | *106780539* | 1.81 | Fructose-bisphosphate aldolase 1 |
|  |  | *106780539* | -2.55 | Fructose-bisphosphate aldolase 1 |  | *106765223* | 1.44 | Fructose-bisphosphate aldolase |
|  |  | *106754931* | -2.18 | Aspartate aminotransferase |  | *106778590* | 1.27 | Phosphoenolpyruvate carboxylase 2 |
|  |  | *106752764* | -1.90 | Glyceraldehyde-3-phosphate dehydrogenase |  | *106762331* | 1.22 | Glyceraldehyde-3-phosphate dehydrogenase B |
|  |  | *106768264* | -1.84 | Ribulose-phosphate 3-epimerase |  | *106762005* | 1.16 | Fructose-bisphosphate aldolase 1 |
|  |  | *106762331* | -1.81 | Glyceraldehyde-3-phosphate dehydrogenase B |  | *106753937* | 1.05 | Glyceraldehyde-3-phosphate dehydrogenase A |
|  |  | *106765223* | -1.79 | Fructose-bisphosphate aldolase |  | *106772878* | 1.82 | NADP-dependent malic enzyme |
|  |  | *106753937* | -1.79 | Glyceraldehyde-3-phosphate dehydrogenase A |  | *106768264* | 1.05 | Ribulose-phosphate 3-epimerase |
|  |  | *106773825* | -1.78 | Ribulose bisphosphate carboxylase small chain 1 |  | *106760805* | 1.76 | Phosphoenolpyruvate carboxylase |
|  |  | *106777824* | -1.67 | Phosphoribulokinase |  |  |  |  |
|  |  | *106754509* | -1.62 | Fructose-1,6-bisphosphatase |  |  |  |  |
|  |  | *106772878* | -1.57 | NADP-dependent malic enzyme |  |  |  |  |
|  |  | *106762005* | -1.44 | Fructose-bisphosphate aldolase 1 |  |  |  |  |
|  |  | *106772585* | -1.42 | Fructose-1,6-bisphosphatase |  |  |  |  |
|  |  | *106770762* | -1.31 | Phosphoenolpyruvate carboxylase 4 |  |  |  |  |
|  |  | *106759537* | -1.29 | Triosephosphate isomerase |  |  |  |  |
|  |  | *106772717* | -1.25 | Fructose-1,6-bisphosphatase |  |  |  |  |
|  |  | *106772090* | -1.18 | Transketolase |  |  |  |  |
|  |  | *106777621* | -1.14 | Glutamate--glyoxylate aminotransferase 2 |  |  |  |  |
|  |  | *106774174* | -1.11 | Ribulose bisphosphate carboxylase small chain 1 |  |  |  |  |
